# Osteohistological signal from the smallest known phytosaur femur reveals slow growth and new insights into the evolution of growth in Archosauria

**DOI:** 10.1111/joa.14185

**Published:** 2024-12-03

**Authors:** Erika R. Goldsmith, Daniel E. Barta, Ben T. Kligman, Sterling J. Nesbitt, Adam D. Marsh, William G. Parker, Michelle R. Stocker

**Affiliations:** ^1^ Department of Geosciences Virginia Tech Blacksburg Virginia USA; ^2^ Department of Anatomy and Cell Biology Oklahoma State University College of Osteopathic Medicine at the Cherokee Nation Tahlequah Oklahoma USA; ^3^ Petrified Forest National Park, Division of Science and Resource Management Petrified Forest Arizona USA; ^4^ Department of Paleobiology, National Museum of Natural History Smithsonian Institution Washington DC USA

**Keywords:** Archosauria, Archosauriformes, growth, ontogeny, osteohistology, Phytosauria

## Abstract

Fossils of embryonic and hatchling individuals can provide invaluable insight into the evolution of prenatal morphologies, heterochronies, and allometric trajectories within Archosauria but are exceptionally rare in the Triassic fossil record, obscuring a critical aspect of archosaurian biology during their evolutionary origins. Microvertebrate sampling at a single bonebed in the Upper Triassic Chinle Formation within Petrified Forest National Park has yielded diminutive archosauriform femora (PEFO 45274, PEFO 45199) with estimated and measured femoral lengths of ~31 mm and ~ 37 mm, respectively. These new specimens provide the unique opportunity to assess the preservation, body size, and growth dynamics of skeletally immature archosauriforms in North America and compare the growth dynamics of archosauromorphs within an evolutionary and ontogenetic context. We assign PEFO 45199 and PEFO 45274 to Phytosauria (Archosauriformes) based on their strongly sigmoidal shape in lateral view, the presence of proximal anterolateral and posteromedial tubera, the absence of an anteromedial tuber of the proximal end, a teardrop‐shaped proximal outline, and a fourth trochanter that is not confluent with the proximal head. Osteohistological analyses of PEFO 45274 reveal a cortex comprising low vascularity, parallel‐fibered bone composed of primary osteons that lacks a hatching line and any lines of arrested growth. We interpret PEFO 45274 as a slow‐growing, post‐hatching individual of less than 1 year of age. Surprisingly, osteohistology of some larger phytosaur femora implies faster growth rates in comparison to PEFO 45274 based on the occasional presence of woven bone and overall higher degrees of vascular density, suggesting the ontogenetic shift from rapid‐to‐slow growth rates might not occur simply or uniformly as expected in Phytosauria and that non‐archosaurian archosauriforms may exhibit size‐dependent histological characteristics. This study highlights the importance of including osteohistology from multiple body sizes to investigate non‐archosaurian archosauriform ancestral growth rates given the phylogenetic position of phytosaurs near the divergence of Archosauria.

## Graphical abstract

The paucity of small, skeletally immature individuals representing the earliest ontogenetic stages of extinct archosaurs complicates our understanding of the growth dynamics within and between species. The opportune finding of the smallest phytosaur femora reveals slow growth, a surprising signal considering that larger phytosaurs in North America have shown evidence of faster growth. This study highlights the importance of size when interpreting osteohistological patterns across Archosauria.

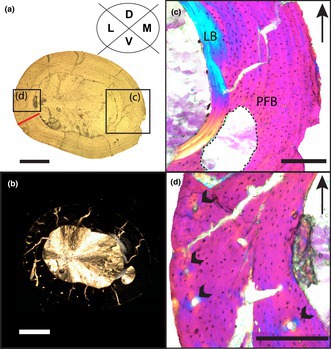

## 1 INTRODUCTION

The initial inspirations for many osteohistological studies in paleontology were investigations into the thermophysiology of dinosaurs and other avian‐line archosaurs (Chinsamy et al., 2012; Chinsamy & Raath, 1992; Chinsamy‐Turan, 2005; Erickson, 2005; Padian et al., 2001; Ricqlès, 1980). This incredible reptile group was shown to display exceptionally fast growth rates and inferred endothermy comparable to those of modern mammals and birds coupled with disparate body sizes (Cubo et al., 2012; Erickson, 2005; Erickson et al., 2009; Legendre et al., 2016; Padian et al., 2001). Even though this finding is consistent with what we know of living avian thermophysiology and metabolism (Chinsamy‐Turan, 2005; de Buffrénil et al., 2021; Wiemann et al., 2022), the other extant reptiles—turtles and tortoises, lepidosaurs, and crocodylians—are known to exhibit slower growth regimes and ectothermy (de Buffrénil et al., 2021). However, in living crocodylians such as *Alligator mississippiensis*, ontogenetically young individuals have been shown to preserve well‐vascularized woven and fibrolamellar bone tissues (Tumarkin‐Deratzian, 2007; Woodward et al., 2014), an indicator of relatively rapid growth early in their life history, which supports phylogenetic and metabolic evidence of rapid ancestral growth rates in archosaurs (Cubo et al., 2012; Legendre et al., 2016).

Osteohistological studies of amniotes also have proven useful towards identifying embryonic tissues in extant and extinct animals, as well as identifying neonatal or hatching lines (i.e., non‐cyclical growth marks that record the moment of birth or hatching, respectively). In living mammals (e.g., *Equus*), embryonic bone tissues have been preserved as zones with largely fibrolamellar complexes (sensu Prondvai et al., 2014) or bone tissues with higher proportions of parallel‐fibered bone in tandem with neonatal lines (Nacarino‐Meneses & Köhler, 2018) that are hypothesized to record the moment of birth (Bruce & Castanet, 2006; Castanet, 2006; Castanet et al., 1993; Castanet & Baez, 1991; Chinsamy & Hurum, 2006; Hugi & Sanchez‐Villagra, 2012). Periosteally to the neonatal lines of *Equus* individuals, the postnatal bone tissue overall increases in vascularity (Nacarino‐Meneses & Köhler, 2018) but may switch to either slow‐growing lamellar bone or more disorganized, woven bone depending on the species (Nacarino‐Meneses & Köhler, 2018). In living reptiles (e.g., *Amblyrhynchus cristatus*), embryonic bone tissue has been documented as monofringent woven‐fibered bone with high degrees of unorganized collagen fibers that grades into parallel‐fibered bone and is abruptly interrupted by a non‐cyclical growth mark interpreted as a hatching line (Hugi & Sanchez‐Villagra, 2012). In the fossil record, hatching lines and embryonic bone have been reported in non‐avian dinosaurs (Barta & Norell, 2021; Curry Rogers et al., 2016; Horner et al., 2001; Reisz et al., 2013; Woodward et al., 2018; Wosik et al., 2020), a small aetosaur humerus (i.e., *Aetosarurus ferratus*; Teschner, Konietzko‐Meier, Desojo, et al., 2022), and a polycotylid plesiosaur (O'Keefe et al., 2019). However, the reported tissue characteristics are variable, reinforcing that a diversity of growth strategies are observed throughout Reptilia, even in the ontogenetically youngest individuals.

Numerous osteohistological analyses of archosaurs (e.g., crocodylians and birds/ dinosaurs) over approximately the last century have revealed an intriguing diversity of growth strategies that have provided valuable insight into skeletochronology (Chinsamy & Raath, 1992; Chinsamy‐Turan, 2005), growth rates (e.g., rapid and slow rates; Amprino, 1947; Case, 1978; Cubo et al., 2012; Dmitriew, 2011; Erickson & Druckenmiller, 2011; Lee et al., 2013; Padian et al., 2001; Padian & Stein, 2013), physiologies (e.g., endothermic and ectothermic conditions; Chinsamy et al., 2012; Erickson & Druckenmiller, 2011), and ecologies (Cruickshank et al., 1996; Hua & de Buffrénil, 1996; O'Keefe et al., 2019; Teschner, Konietzko‐Meier, & Klein, 2022). However, investigations of the evolution of these growth regimes and nuanced comparisons of archosaurian growth strategies have only been conducted within the last two decades (Botha‐Brink & Smith, 2011; Cubo et al., 2012; de Ricqlès et al., 2003; Klein et al., 2017; Legendre et al., 2016; Werning, 2013; Werning & Nesbitt, 2016) and are hindered by a lack of extensive phylogenetic and ontogenetic osteohistological sampling due to specimen availability (Botha‐Brink & Smith, 2011). This is especially true of clades positioned near the base of the archosaur phylogeny, which may provide valuable information about the divergence and evolution of these diverse growth strategies (Botha‐Brink & Smith, 2011; Werning, 2013).

Studying the growth of organisms that form the successive outgroups to Archosauria (i.e., non‐archosauriform archosauromorphs and non‐archosaurian archosauriforms) may provide additional data on ancestral growth rates in saurian reptiles overall (Werning & Nesbitt, 2016). Osteohistological studies of non‐archosauriform archosauromorphs (e.g., *Prolacerta* and rhynchosaurs; Botha‐Brink & Smith, 2011; Werning & Nesbitt, 2016) revealed parallel‐fibered bone with simple vascular canals arranged within a longitudinal network that indicated slower growth rates than those of archosauriforms and archosaurs (Botha‐Brink & Smith, 2011; Legendre et al., 2016; Werning & Nesbitt, 2016). Archosauriformes were reported to preserve moderately vascularized woven bone tissue that is weakly fibrolamellar (e.g., *Proterosuchus*; Botha‐Brink & Smith, 2011), highly vascularized fibrolamellar bone complexes (e.g. *Erythrosuchus*; Botha‐Brink & Smith, 2011), or highly vascularized parallel‐fibered bone (e.g., *Euparkeria*; Botha‐Brink & Smith, 2011) suggesting faster growth rates. Despite the additions of these taxa into the osteohistological database, the majority of these studies were conducted on the largest representative individuals of those species. Therefore, we cannot assess how the growth regime may have changed throughout ontogeny to determine if ancestral growth patterns may have been preserved during the earliest growth stages in those groups, similar to what is commonly seen in living crocodylians (Tumarkin‐Deratzian, 2007; Woodward et al., 2014, 2011).

Given their phylogenetic position near the base of Archosauria, the Phytosauria (large semi‐aquatic archosauriforms known from Middle Triassic to Upper Triassic deposits globally; Stocker & Butler, 2013; Stocker et al., 2017) provide a unique opportunity to study the diversification and evolution of growth dynamics at the divergence of pseudosuchian and avemetatarsalian archosaurs. Phytosaurs are considered either non‐archosaurian archosauriforms and the sister‐taxon of Archosauria (Nesbitt, 2011) or the earliest‐diverging pseudosuchian archosaurs (Benton & Clark, 1988; Ezcurra, 2016; Juul, 1994; Parrish, 1993; Sereno, 1991). Osteohistological analyses of phytosaur specimens have revealed moderate to low degrees of vascularity within both parallel‐fibered and woven bone tissues (Butler et al., 2019; de Ricqlès et al., 2003; Teschner, Konietzko‐Meier, & Klein, 2022) similar to what is reported for other archosauriforms (e.g., *Euparkeria*; Botha‐Brink & Smith, 2011; Legendre et al., 2013) and some pseudosuchians (e.g., Crocodylia; de Andrade et al., 2020; Mascarenhas‐Junior et al., 2021; Tumarkin‐Deratzian, 2007; Woodward et al., 2011, 2014). However, phytosaur osteohistology to date has not included earlier growth stages mainly because of the absence of skeletally immature phytosaur skeletons and the paucity of easily identifiable small specimens known from isolated bones.

Here we report the smallest known phytosaur femora, found via extensive microvertebrate collection efforts at PFV 456 (the “Thunderstorm Ridge” locality) in the lower Chinle Formation at Petrified Forest National Park (PEFO). This unique discovery fills a gap in our understanding of the ontogenetic size distribution of phytosaurs and allows us to assess (1) whether these specimens may belong to embryonic or hatchling individuals, (2) the body size estimate at that femoral size and hypothesized ontogenetic stage, (3) the growth dynamics of a diminutive phytosaur, and (4) the hypothesized growth strategies of phytosaurs in the context of archosaurian evolution. With these data, we compare the anatomy and growth dynamics of these PEFO specimens to those of larger phytosaurs, pseudosuchians, and archosauriforms to investigate whether there are shared growth strategies among these taxa.

Institutional Abbreviations: BSP, Bayerische Staatssammlung für Paläontolgie und Geologie, Munich, Germany; FMNH, Field Museum of Natural History, Chicago, Illinois, U.S.A.; GR, Ghost Ranch Ruth Hall May Museum of Paleontology, New Mexico, U.S.A.; ISI, Indian Statistical Institute, Kolkata, India; MCNSB, Museo Civico di Scienze Naturali “E. Caffi” di Bergamo, Lombardy, Italy; MCN‐PV, Museu de Ciências Naturais – Fundação Zoobotânica do Rio Grande do Sul, Porto Alegre, Brazil; MCZ, Museum of Comparative Zoology, Cambridge, Massachusetts, U.S.A.; MOR‐OST, Museum of the Rockies Osteology collections, Bozeman, Montana, U.S.A.; NHMUK, Natural History Museum, London, United Kingdon; NHMW, Naturhistorisches Museum Wien, Vienna, Austria; PEFO, Petrified Forest National Park, Petrified Forest, Arizona, U.S.A.; PVL, Paleontología de Vertebrados, Instituto “Miguel Lillo,” San Miguel de Tucumán, Argentina; SAM‐PK, Iziko‐South African Museum, Cape Town, South Africa; SMNS, Staatliches Museum für Naturkunde Stuttgart, Stuttgart, Germany; TTU P, Texas Tech Museum of Paleontology, Lubbock, Texas, U.S.A.; UCMP, University of California Museum of Paleontology, Berkeley, California, U.S.A.; UOPB, University of Opole, Institute of Biology, Laboratory of Palaeobiology, Opole, Poland; USNM, National Museum of Natural History, Smithsonian Institution, Washington, D.C., U.S.A.; UWBM, Burke Museum of Natural History and Culture, University of Washington, Seattle, Washington, U.S.A.

## 2 METHODS

### 2.1 Field collection and preparation

**FIGURE 1:**
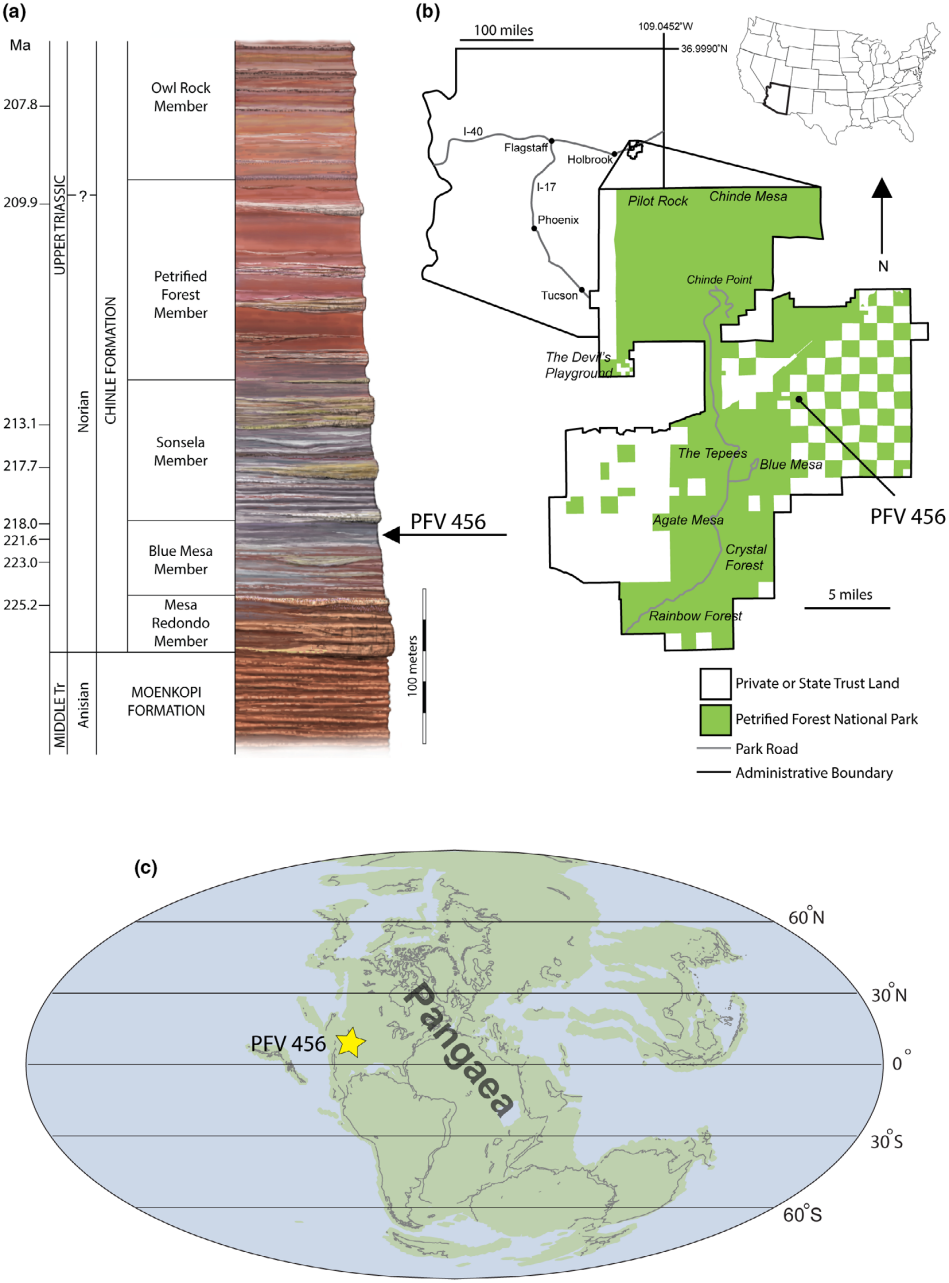
Stratigraphic (a, modified from Martz et al. [2012]), geographic (b), and paleogeographic (c) positions of the Thunderstorm Ridge locality (PFV 456) in the Petrified Forest National Park.

PEFO 45199 and PEFO 45274 were collected in 2022 by the PEFO paleontology field team from PFV 456 (Thunderstorm Ridge locality) within the upper Blue Mesa Member, Chinle Formation, near the Puerco River in PEFO, Arizona, USA (Figure 1a,b). The Blue Mesa Member of the Chinle Formation was deposited in a northwest‐flowing fluviolacustrine system in equatorial Pangaea (Figure 1a–c) in a humid climatic setting (e.g., Lepre & Olsen, 2021). These specimens were collected using the same field and lab microvertebrate screenwashing methodologies described by Kligman et al. (2023). The disarticulated, and often delicate, skeletal elements found in the PFV 456 bonebed likely were deposited initially in a lacustrine setting and were later reworked and deposited in their final position by a high‐energy channel avulsion event (Kligman, 2023). The composition of the vertebrate assemblage captures a community living in a marginal lacustrine environment (Kligman, 2023), and includes chondrichthyans, actinopterygians, actinistians, dipnoans, lissamphibians (Kligman et al., 2023), metoposaurid temnospondyls, drepanosauromorphs (Jenkins et al., 2020), lepidosauromorphs, non‐archosaur archosauromorphs (e.g., Marsh et al., 2024; Mellett et al., 2023), phytosaurs (e.g., Kligman, 2023), pseudosuchians (e.g., Marsh et al., 2020), ornithodirans (e.g., Marsh & Parker, 2020), and cynodonts (Kligman et al., 2020). In addition to the unusually high species richness of PFV 456 (e.g., Kligman, 2023), PFV 456 opens a rare window into the ontogeny of Triassic continental vertebrates. Phytosaurs are one of many vertebrate taxa represented by growth series at PFV 456, and this study suggests the potential to explore the early ontogeny of other Triassic groups where fossils of early‐stage individuals are rare or unknown.

### 2.2 Micro‐computed tomography (μCT)

Prior to destructive osteohistological sampling, PEFO 42754 and PEFO 45199 were photographed and μCT scanned to preserve their external and internal morphology at the Virginia Tech Institute for Critical Technology and Applied Science (ICTAS) using a Skyscan 1172 Microfocus X‐radiographic Scanner. PEFO 45274 was scanned at 91 kV and 110 μA with a copper and aluminum filter and an exposure time of 2904 ms. The resulting scan produced 838 projections with a pixel size of 13.76 μm. PEFO 45199 was scanned at 92 kV and 108 μA with a copper and aluminum filter and exposure time of 4840 ms. That resulting scan produced 739 projections with a pixel size of 16.03 μm.TIFF files were post‐processed using Avizo Lite 2019.3 3D software, and bone was segmented using the thresholding tool to produce a 3D digital model. High‐resolution μCT scans and 3D models are available for download on Morphosource.org (Project ID #000628894).

### 2.3 Osteohistological preparations

**FIGURE 2:**
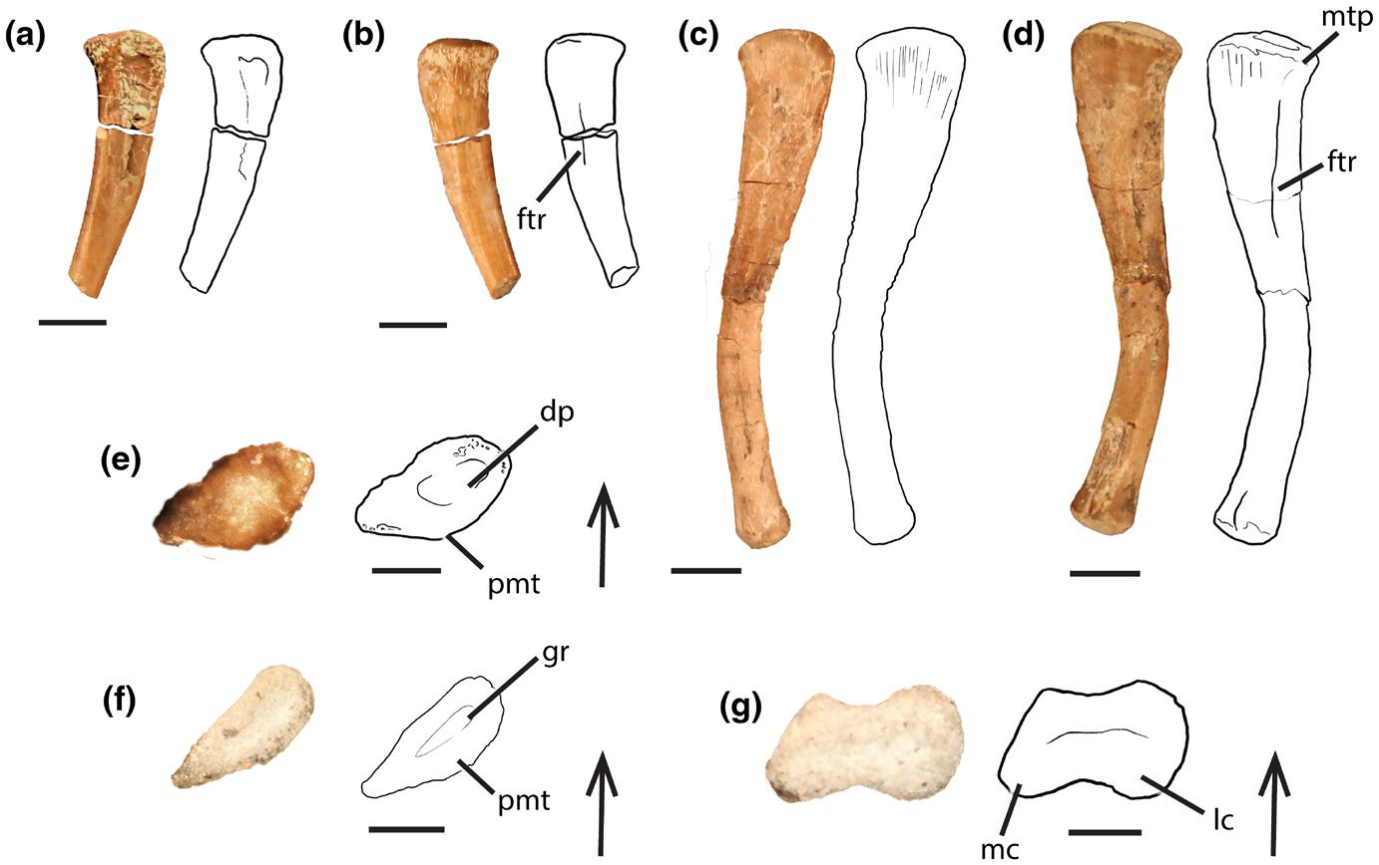
Diminutive phytosaur femora from the “Thunderstorm Ridge” locality (PFV 456) in the lower Chinle Formation at Petrified Forest National Park. Left femur (PEFO 45274) in lateral (a), medial (b), and proximal (e) views; Left femur (PEFO 45199) in lateral (c), medial (d), proximal (f) views, and distal (g) views; Scale bars = 5 mm; Arrows point anteriorly; Dp, depression; ftr, fourth trochanter; gr, groove; mtp, metaphyseal line; pmt, posteromedial tuber.

To determine the skeletochronology and bone vascular characteristics of a minuscule phytosaur femur, we conducted osteohistological analyses of PEFO 42574 because this specimen was already fragmentary and incomplete (missing the distal half of the femur) when collected (Figure 2a,b); We selected PEFO 42574 for histological analyses to maintain the preservation of the smallest, complete phytosaur femur (i.e., PEFO 45199) in its entirety. We followed the methodology of Chinsamy and Raath (1992) and Lamm (2013) using the equipment in the Fossil Preparation Lab at Virginia Tech in Blacksburg, Virginia, USA. Even though it has been observed that osteohistological thin section methods (i.e., petrographic versus microtome methods) produce variable skeletochronological results that may result in the underestimation of LAG counts in fossil material (Schucht et al., 2021), microtome methodology is not possible in any paleohistological study due to the dense, brittle, and inflexible nature of fossils (Wilson, 1994). The femoral midshaft region of PEFO 42754 was taphonomically broken and separated from the proximal end. The midshaft section of interest was entirely embedded in Eager Polymers Crystal Clear Polyester Resin (EP4101UV). After the resin cured, we cut a transverse 1.5 mm wafer with a Buehler IsoMet® 1000 Precision Saw equipped with a diamond‐tipped blade and used Loctite cyanoacrylate glue to mount the wafer onto a frosted plexiglass slide. The section was ground on a Buehler Ecomet III grinder/polisher using a sequence of progressively finer grit paper (400–2400 grit) until the wafer was thin enough to observe histological features under a petrographic microscope. Histological features were observed and documented using an Olympus BX51 microscope equipped with a Lumenera Infinity 1 digital camera. High‐resolution photomicrographs of PEFO 45274 are also available for download on Morphosource.org (Project ID #000628894).

### 2.4 Body size estimation

To determine the baseline size by which we can compare all phytosaur growth and body size studies to gain detailed insight into the rate at which phytosaurs grew, we calculated the body sizes of both PEFO 45199 and PEFO 45274. Body size and mass estimation in phytosaurs has been largely untested with only a single study that used phytosaur cranial and limb dimensions to infer total length (TL) and body mass (BM) (Hurlburt et al., 2003). Body size calculations in phytosaurs are complicated by the fact that there are only two known nearly complete skeletons of phytosaurs (*Parasuchus hislopi* [ISI R 42]; Chatterjee, 1978 and *Mystriosuchus* sp. [MCNSB 10087]; Gozzi and Renesto, 2003) that preserve the total body proportions, including the tail. However, only MCNSB 10087 has the caudal length reported (~1920 mm; Gozzi and Renesto, 2003), which comprises 70–75 caudal vertebrae that make up 50% of the total length (~3760 mm; Gozzi and Renesto, 2003) of the individual. In comparison, *Alligator* tails possess only 34 caudal vertebrae (Hurlburt et al., 2003) but also are 50% of the total length because *Alligator* snout‐vent length (SVL) is approximately 50% of TL (Hurlburt et al., 2003; Woodward et al., 1995). However, it is important to note the relationship between tail length and total length in phytosaurs was based on a single specimen of a single species of phytosaur with an inferred (and unique amongst phytosaurs) marine ecology and that estimate may be heavily influenced by the species' unique ecology, rather than a trait shared by all of Phytosauria. Therefore, it is possible that using *Alligator* size as a priori data for regressions may underestimate predicted phytosaur TL. To combat this potential underestimation, we also calculated SVLA (the anterior extent of the SVL using the posterior extent of the ischium as a skeletal proxy) from Hurlburt et al. (2003) and body mass (BM) using the data from Campione and Evans (2012) in tandem with TL.

We measured both femora using digital calipers and specifications provided by Farlow et al. (2005) (Table 1). Measured and estimated femoral lengths (Table S1; FL; mm) were used to calculate TL (Table S2; mm) of PEFO 45199 and all comparative taxa (Table S1) with the following equation from Farlow et al. (2005):

(1) $$\mathrm{TL}=14.45\;\left(\mathrm{FL}\right)+1.45\;\left(p=<0.001;R^{2}=0.996;95\%\mathrm{CI}=\text{not reported}\;\left[\mathrm{NR}\right]\right)$$

**TABLE 1:** Femoral measurements (mm) of phytosaurs used for comparative anatomy based on dimensions from Farlow (2005).

Specimen number	FL	Fc	Ftr	Fpmn	Fpmx	Fdw	Fdh
PEFO 45199	34.12	11	12.28	3.24	7.52	6.4	3.44
PEFO 45274	NA	6.47	9.3	2.5	4.72	NA	NA
AMNH FR 32182	174	65	58.36	19.59	52.91	38.24	22.02
UCMP 27200	476^a^	164	161	53.42	126.03	107.23^a^	76.61^a^

Abbreviations: Fc, femoral circumference; Fdh, height of distal condyles; Fdw, width of distal condyles; FL, femoral length; Fpmn, femoral head anterior–posterior length; Fpmx, femoral head mediolateral width; Ftr, distance between femoral head and fourth trochanter.

^a^ Measurements include reconstructions.

Because it is incomplete, the TL (mm) of PEFO 45274 was determined using the distance between the proximal head and the fourth trochanter (Ftr; mm) and the following equation from Farlow et al. (2005):

(2) $$\text{logTL}=0.88\;\left(\text{logFtr}\right)+1.81\;\left(p=<0.001;R^{2}=0.99;95\%\mathrm{CI}=\mathrm{NR}\right)$$

However, the utility of Equation 2 to predict TL in skeletally immature specimens may be complicated by the ontogenetic distal migrations of the fourth trochanter that have been observed in some archosaurs (Griffin et al., 2019); therefore, we also employed the following equation from Campione and Evans (2012) to reconstruct femoral lengths from femoral circumferences (Fc; mm). The equations from Campione and Evans (2012) provided a broader taxonomic range that can be utilized across all of Reptilia and found that:

(3) $$\text{logFL}=\frac{\text{logFc}+0.8115}{1.1751}\;\left(0.1<p>0.05;R^{2}=0.86;95\%+\mathrm{CI}=-0.5884\;\text{to}-1.0347\right)$$

Femoral circumferences were either measured in‐person using a seamstress tape measure or using osteohistological images and the measure tool in the FIJI image analysis software (Schindelin et al., 2012) that determined the perimeter of an outlined feature. After all femoral lengths from circumferences were attained, we used Equation 1 to determine all total lengths.

We estimated the femoral length of UCMP 29251 utilizing bone cortex thin section images from Werning (2013) and assumptions about the thickness of the medullary cavity with similar cortical growth because the size of the element was never reported, nor are there photomicrographs available of the entire thin section to measure femoral circumference to use in Equation 3. Therefore, we used the open‐source software R (https://www.r‐project.org/) to create a linear regression to predict phytosaur femoral length using the maximum midshaft widths (MMSW) from phytosaurs (*n* = 12; Table S1) which produced the following equation:

(4) $$\begin{array}{c} \text{logFL}=0.92\text{logMMSW}+1.03\;\left(p=<0.001;R^{2}=0.98;95\%\mathrm{CI}=0.83\;\text{to}\;1.03\right) \end{array}$$

We estimated the MMSW of UCMP 25291 (MMSW; Werning, 2013) by doubling the cortical thickness reported by Werning (2013) and adding the measured maximum width of the medullary cavity (using FIJI imaging analysis software; Schindelin et al., 2012) of UOPB 01026 (*Parasuchus* cf. *arenaceus*; Teschner, Konietzko‐Meier, Desojo, et al., 2022; Teschner, Konietzko‐Meier, & Klein, 2022) which preserved a similar cortical thickness to UCMP 25291 (~ 6–9 mm).

To account for the possibility that phytosaur TL may be underestimated using femoral data due to the unknown tail lengths across Phytosauria, we also calculated the SVLA (mm; Hurlburt et al., 2003; Farlow et al., 2005). The SVLA of PEFO 45199 was calculated using the following equation (Hurlburt et al., 2003):

(5) $$\text{SVLA}=7.17\;\left(\mathrm{FL}\right)+5.83\;\left(p=\mathrm{NR};R^{2}=0.99;95\%\mathrm{CI}=\mathrm{NR}\right)$$

The SVLA of PEFO 45274 was calculated by using Equation (3) to estimate FL (mm) and then utilizing the estimated FL value to estimate SVLA in Equation (5) above.

Lastly, we calculated the BM (g) of PEFO 45199 and PEFO 45274 from measured and estimated FL (mm), respectively, using the following equation from Campione and Evans (2012):

(6) $$\text{logBM}=\log3.25\mathrm{FL}-2.48\;\left(0.05<p>0.01;R^{2}=0.78;95\%\mathrm{CI}=-1.71\;\text{to}-3.25\right)$$

All calculations for specimen estimates in this study and any comparative taxa were calculated in Excel and are reported in Tables S1 and S2.

### 2.5 Growth rate calculation

To test whether PEFO 45274 represents a skeletally immature individual or an older, yet still small, individual, we followed the approach of Barta et al. (2022; see their supplementary information) to calculate growth rate. Body size does not always equate to skeletal maturity (Griffin et al., 2020), and it is possible that individuals may grow continuously without depositing annual lines of arrested growth (LAGs) during a multi‐year life‐span, though other cyclical growth marks may be present (Teschner, Konietzko‐Meier, & Klein, 2022). This calculation tests whether the preserved thickness of cortical bone is too great to have been plausibly deposited within a single year (i.e., representing multi‐year growth uninterrupted by LAGs or annuli). If the estimated growth rate of PEFO 45274 greatly exceeds the growth rates estimated for other extinct archosauromorphs (Cubo et al., 2012), or the experimentally derived growth rates for the femora of a variety of extant amniotes (Cubo et al., 2012), or the long bones of extant mallard ducks (*Anas platyrhynchos*) (de Margerie et al., 2002), then this implausibly high growth rate would suggest that PEFO 45274 instead represents a more skeletally mature individual, potentially falsifying our hypothesis that this specimen may belong to a hatchling individual. Alternatively, if the growth rate of PEFO 45274 does not greatly exceed the comparative rates, then deposition of the preserved bone entirely within a year (albeit not necessarily the first year of life) remains a viable hypothesis, and a lack of annual growth marks would be less surprising. However, such a result by itself is not unequivocal positive evidence for hatchling status of the specimen because the preserved bone could still signify slow, possibly continuous, growth during a single or multiple post‐hatchling years.

We measured the cortical thickness of PEFO 45274 to calculate a rough estimate of its daily appositional growth rate to assess how plausible this growth rate is compared to femoral growth rates of related taxa as described above. We used FIJI image analysis software (Schindelin et al., 2012) to measure a transect through the lateral quadrant of the femur to capture the preserved cortical thickness from the medullary cavity to the periosteal surface. The choice of measurement location was arbitrary, except that we wished to avoid any areas that may incorporate non‐uniform growth rates (e.g., endosteal lamellar bone signifying cortical drift; de Margerie et al., 2002). The calculated values are necessarily underestimates of the growth rate because it is probable that some of the cortex had been removed by medullary cavity erosion prior to the animal's death. Following Barta et al. (2022), we calculated hypothetical growth rates for hypothetical continuous yearly growth, using the 381‐day Triassic year of Wells (1963) and years that include 90‐day (e.g., Woodward et al., 2015) and 190.5‐day (half year) growth hiatuses.

## 3 RESULTS

### 3.1 Systematic paleontology

ARCHOSAURIFORMES Gauthier et al., 1988.

PHYTOSAURIA von Jäger, 1828 sensu Doyle & Sues, 1995.

Referred Specimens—PEFO 45274, an incomplete left femur missing its distal half (Figure 2a,b,e), and PEFO 45199, a complete left femur (Figure 2c,d,f,g).

Description and Rationale for Taxonomic Assignment—Phytosaur femora are able to be assigned only to higher clade level, not genus or species, using a combination of morphological character states (detailed below) because assignment of skeletal material to genus‐level taxa within Phytosauria currently is solely dependent upon cranial features (Butler et al., 2019; Hungerbühler, 2002; Hungerbühler et al., 2013; Jones & Butler, 2018; Stocker, 2010; Stocker et al., 2017; Stocker & Butler, 2013) due to the paucity of associated and articulated skeletons and prevalence of cranial material in the published fossil record. As a result, there currently is no framework to taxonomically identify isolated phytosaur postcrania to a particular phytosaur taxon. However, taxonomic assignments to the least‐inclusive clades are possible and are important for establishing the presence of groups in the fossil record (e.g., Bell et al., 2010; Bell & Mead, 2014; Bever, 2005; Lessner et al., 2018; Nesbitt & Stocker, 2008); therefore, we employ apomorphy‐based methodologies in combination with absence of character states to assign the two specimens in this study to the least‐inclusive clade.

PEFO 45199 is approximately 34 mm in length. In lateral and anterior views, PEFO 45199 is sigmoidal in shape, and the angle between the femoral head and the transverse plane of the distal condyles is approximately 90° (Figure 2c,d), a trait that can be observed in *Prolacerta broomi*, *Erythrosuchus africanus*, *Proterosuchus fergusi*, *Vancleavea campi*, and phytosaurs (Nesbitt, 2011). The lateral distal condyle projects further distally than the medial condyle, which is apomorphic of allokotosaurians, *P. broomi*, phytosaurs, and pseudosuchian archosaurs (Lessner et al., 2018). The surface between the femoral condyles is smooth (Figure 2b; Nesbitt, 2011: character 322:0; Ezcurra, 2016: character 514:0), a feature observed in *P. broomi, P. fergusi, E. africanus*, non‐archosaurian archosauriforms, phytosaurs, and aetosaurs. However, the distal condyles of PEFO 45199 do not dorsoventrally project or expand beyond the extent of the shaft (Figure 2c,d; Nesbitt, 2011: character 318:1; Ezcurra, 2016: character 511:1) in contrast to the prominent expansions observed in *P. broomi*, *E. africanus*, allokotosaurians (e.g., *Trilophosaurus buettneri*) and rhynchosaurs (Nesbitt, 2011: character 318:0; Ezcurra, 2016, character 511:0). Therefore, the lack of expanded distal condyles observed in PEFO 45199 allows for the taxonomic assignment to Archosauriformes (e.g., phytosaurs, *V. campi*, *Euparkeria capensis*). Additionally, the fourth trochanter of PEFO 45199 is long, strap‐like, broad and positioned on the proximal one‐third of the shaft such that the trochanter is not close to the proximal head (Figure 2d). This condition is apomorphic of archosauriforms (Nesbitt, 2011: character 315:1; Ezcurra, 2016: character 504:2) and allows for taxonomic assignment to Archosauriformes (e.g., phytosaurs) as well as Archosauria and precludes assignment to non‐archosauriform archosauromorphs. The assignment of PEFO 45199 to Archosauriformes is further supported by the presence of anterolateral and posteromedial tubera on the femoral head, which, in proximal view, preserves a “tear‐drop”‐shaped outline with narrow posterolateral edges (Figure 2f) accompanied by the lack of an anteromedial tuber (=posteromedial tuber from Ezcurra, 2016), suggesting a taxonomic assignment to non‐archosaurian archosauriforms (Nesbitt, 2011). However, the femora of archosaurs, including aetosaurs, *Revueltosaurus callenderi* (e.g., PEFO 34561; Nesbitt, 2011), ornithosuchids, dinosauromorphs, and crocodylomorphs possess three proximal tubera (i.e., anterolateral, anteromedial, and posteromedial tubera [=anterior, posteromedial, and posterior tubera, respectively, from Ezcurra, 2016]), therefore preventing taxonomic assignment of PEFO 45199 to most clades within Archosauria and possibly to the whole group Archosauria. The “tear‐drop”‐shaped proximal surface of PEFO 45199 differs in comparison with the elongate oval shape of the proximal end of the femur of *V. campi* (GR 138; Nesbitt et al., 2009), precluding taxonomic assignment to *V. campi*. Therefore, based on the character states listed above, we assign PEFO 45199 to Phytosauria. The presence of a proximal anteromedial tuber (Nesbitt, 2011: character 300:1) is diagnostic of pseudosuchian and avemetatarsalian archosaurs. Therefore, the lack of this tuber in phytosaurs was considered to be apomophic of phytosaurs and helped provide the basis for their diagnosis as non‐archosaurian archosauriforms (Nesbitt, 2011). However, we recognize that the utility of the lack of an anteromedial tuber as an apomorphy for Phytosauria (Nesbitt, 2011) was contested by Ezcurra (2016) due to his observation of this feature in *Parasuchus hislopi* (ISI R42; Ezcurra, 2016). Interestingly, Ezcurra (2016) did not observe anteromedial tubera in either *Nicrosaurus kapffi* (SMNS 4381/1,2) or *Smilosuchus gregorii* (USNM 18313) and ultimately considered the lack of an anteromedial tuber synapomorphic to mystriosuchin (=pseudopalatine) phytosaurs. Therefore, the lack of an anteromedial tuber may be apomorphic for Adamanian holochronozone leptosuchomorph phytosaur taxa, the only phytosaur clade currently known from the Chinle Formation at Petrified Forest National Park (Parker & Martz, 2010). Additionally, phytosaurs are well‐represented throughout the Petrified Forest National Park and specifically at PFV 456 (e.g., PEFO 46437 [Parasuchidae premaxilla], PEFO 49717 [Parasuchidae partial opisthotic], PEFO 44698 [Parasuchidae proximal end of scapula], PEFO 49399 [Parasuchidae partial frontal], and PEFO 44705 [Parasuchidae appendicular osteoderm]) and upper Blue Mesa Member deposits (e.g., PEFO 46906/UWBM 118800 [*Smilosuchus*, mostly complete skull]). Therefore, assignment of the isolated PEFO 45199 from PFV 456 to Phytosauria is well‐supported based on the shared morphologies with other phytosaur femora and the known presence of copious isolated phytosaur elements from PFV 456 and coeval upper Blue Mesa Member localities.

PEFO 45274 only preserves its proximal 14.8 mm (~half of its hypothesized total length), including the proximalmost extent of the fourth trochanter. What is present of the fourth trochanter of PEFO 45274 is consistent with the condition preserved in PEFO 45199, in which the proximalmost extent is well distal of the proximal surface of the femoral head (Figure 2b), a character state diagnostic of archosauriforms (Ezcurra, 2016; Nesbitt, 2011). The femoral head of PEFO 45274 also possesses anterolateral and posteromedial tubera that, in proximal view, outline a “tear‐drop” shape. Similarly to PEFO 45199, it preserves narrowed posterolateral edges and lacks an anteromedial tuber (Figure 2e), which similarly allows a taxonomic assignment to non‐archosaurian archosauriforms. The lack of an anteromedial tuber in PEFO 45274 also prevents taxonomic assignment to *V. campi*, pseudosuchian archosaurs (e.g., crocodylomorphs, Aetosauria, and *Postosuchus*), and avemetatarsalian archosaurs (e.g., *Silesaurus*, lagerpetids, *Dromomeron*, and coelophysoids) (Nesbitt, 2011: character 302:1). Again, the “tear‐drop”‐shaped proximal surface in proximal view (Figure 2e) drastically differs in comparison to the elongate and oval shape of the proximal surface of a femur of *Vancleavea campi* (GR 138; Nesbitt et al., 2009), adding further evidence to exclude these femora from assignment to *Vancleavea*. Based on the combination of character states here described, we also assign PEFO 45274 to Phytosauria. There are currently no femoral apomorphies to differentiate any of the Adamanian leptosuchomorph phytosaurs from the Blue Mesa Member of the Chinle Formation (nor any Triassic phytosaurs), so we cannot attribute them to a less inclusive clade than Phytosauria.

### 3.2 Potential ontogenetic features

**FIGURE 3:**
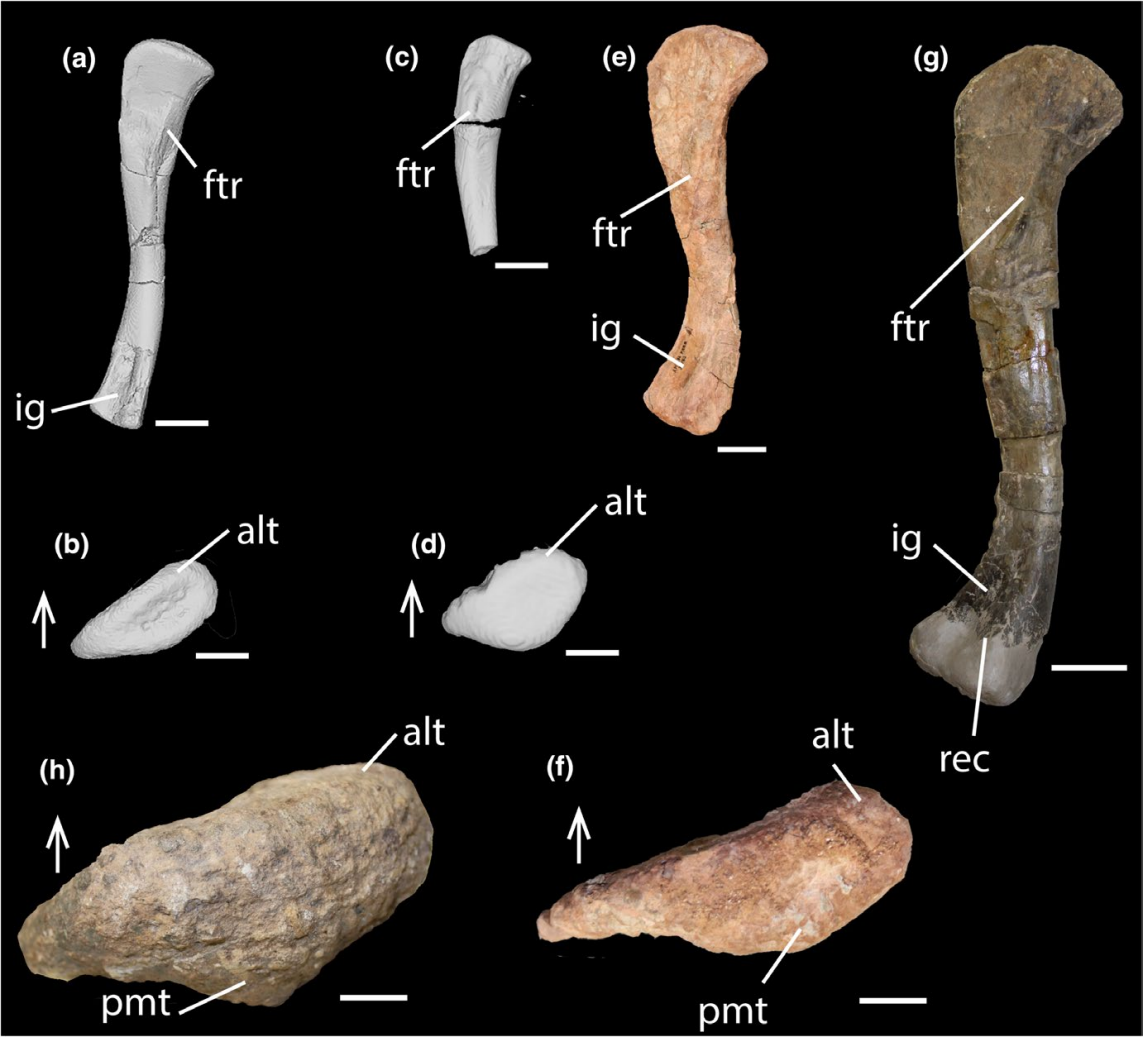
Comparative anatomy of phytosaur femora of various sizes. Left femur of PEFO 45199 in medial (a; scale bar = 5 mm) and proximal (b; scale bar = 5 mm) views. Left femur of PEFO 45274 in medial (c; scale bar = 5 mm) and proximal views (d; scale bar = 5 mm). Left femur of an inferred skeletally immature mystriosuchin phytosaur (AMNH FR 32182) in medial (e; scale bar = 2 cm) and proximal (f; scale bar = 1 cm) views. Left femur of *Smilosuchus gregorii* (UCMP 27200) in medial (g; scale bar = 5 cm) and proximal views (h; scale bar = 1 cm). Arrows point anteriorly. Alt, anterolateral tuber; ftr, fourth trochanter; ig, intercondylar groove; pmt, posteromedial tuber; rec, reconstruction.

Despite the shared features between PEFO 45199 and PEFO 45274 and larger phytosaur femora, shape differences are present and potentially attributable to ontogenetic transformations. The proximal margin of the femoral head of PEFO 45199 in anterior and posterior views (Figure 2c,d; Figure 3a) and of PEFO 45274 (Figure 2a,b; Figure 3c) is relatively flattened, with PEFO 45274 preserving a flatter surface than PEFO 45199. Typically, this margin is more rounded in larger phytosaur femora (FL > 200 mm; e.g., *Smilosuchus gregorii*, UCMP 27200 [Table 1; Figure 3g–h]; *Parasuchus hislopi*, ISI 42 [Chatterjee, 1978: text‐figure 13]; *Mystriosuchus steinbergeri*, NHMW 1986/0024/0012 [Butler et al., 2019]). However, a femur belonging to a skeletally immature mystriosuchin parasuchid, AMNH FR 32182 (Figure 3e; FL = 180 mm; Table 1; Goldsmith et al., 2023) shows an intermediate condition between PEFO 45199 and PEFO 45274, and UCMP 27200 (i.e., more rounded than PEFO 45199 or PEFO 45274, yet less rounded than UCMP 27200), suggesting that this difference may be attributable to ontogeny. In living reptiles, epiphyseal ossification can occur where there may be thicker cartilage in skeletally immature and smaller individuals relative to their larger, skeletally mature counterparts (Collett, 2023; Griffin et al., 2020; Tsai & Holliday, 2015). In extinct taxa, the cartilage cap of skeletally immature femora generally does not preserve, creating a more flattened, though often rugose, ossified edge in comparison to larger femora with ossified proximal ends (Collett, 2023; Holliday et al., 2010; Tsai & Holliday, 2015).

The flatter proximal surface of PEFO 45199 and PEFO 45274 also preserves a transverse and straight groove (Figures 2f, 3b; character 314:1; Nesbitt, 2011) and a shallow central depression (Figure 2e), respectively, as opposed to the smooth surface typically seen in larger phytosaur femora (e.g., *S. gregorii*, UCMP 27200; Figure 3h) and most non‐archosaurian archosauriforms (Nesbitt, 2011: text‐figure III.25). A transverse groove in the proximal surface of the femoral head is a trait observed in some non‐archosaurian archosauriforms (i.e., *E. africanus* [Gower, 2003; Nesbitt, 2011] and *Chanaresuchus bonapartei* [MCZ 4035; Nesbitt, 2011]), as well as some pseudosuchian archosaurs (i.e. *Aetosauroides scagliai* (PVL 2073), small individuals of *Typothorax coccinarum* from the Canjilon Quarry, New Mexico [Nesbitt, 2011], *Arizonasaurus babbitti* [MSM 4596], *Effigia okeeffeae* [AMNH FR 30588], *Shuvosaurus inexpectatus* [TTU P‐9001; Nesbitt & Chatterjee, 2024], *Poposaurus gracilis* [FMNH UR 357], *Prestosuchus chiniquensis* [BSP XXV 1–3/5–11/28–41/49; Nesbitt, 2011], *Batrachotomus kupferzellensis* (SMNS 52970; Nesbitt, 2011)). If PEFO 45274 and PEFO 45199 do belong to skeletally immature phytosaur individuals, the presence of a depression or groove on the proximal surface of small phytosaur femora may be caused by a cartilage cone that is observed to become shallower and less visible through growth in extant crocodylians (Tsai & Holliday, 2015) and early theropods (Griffin, 2018), and may denote skeletal immaturity in Phytosauria. However, no other phytosaur femora less than 100 mm in length are known with which to test this finding, and it is unclear whether this may be an ontogenetic feature, expected morphological variation, or potentially, a diagnostic feature of a different non‐phytosaurian taxon.

The fourth trochanter of PEFO 45274 is much less extended from the shaft in comparison to that of PEFO 45199 (Figure 3a,c). However, both are significantly less extended from the shaft in comparison to larger phytosaur femora (e.g., *S. gregorii*, UCMP 27200; FL ≈480 mm; Figure 3h). We hypothesize that the shape of the fourth trochanter changes through ontogeny in phytosaurs similar to what was reported for *Dromomeron gregorii* (Griffin et al., 2019; Nesbitt et al., 2009) such that the ridge extends away from the shaft through growth and the proximal half appears more thin and ridge‐like than the distal half. In contrast to *D. gregorii* femoral ontogeny, phytosaurs may attain a fourth trochanter that extends further early in ontogeny (i.e., before reaching 34 mm in femoral length), and potentially (similarly to *A. mississippiensis*; [Dodson, 1975, Livingston et al., 2009]) may always preserve a fourth trochanter postnatally. It is still unclear whether the fourth trochanter in phytosaurs migrates distally through ontogeny in a way similar to what was reported for *A. mississippiensis* (Dodson, 1975; Livingston et al., 2009). Based on the size series of phytosaur femora discussed in this study, the fourth trochanter does not appear to migrate distally through ontogeny (Figure 3a,c,e,g). However, the extremely limited sample size of phytosaur femora currently representing an ontogenetic series hinders our ability to test the extent of fourth trochanter shape change through ontogeny.

### 3.3 Osteohistology and microanatomy

**FIGURE 4:**
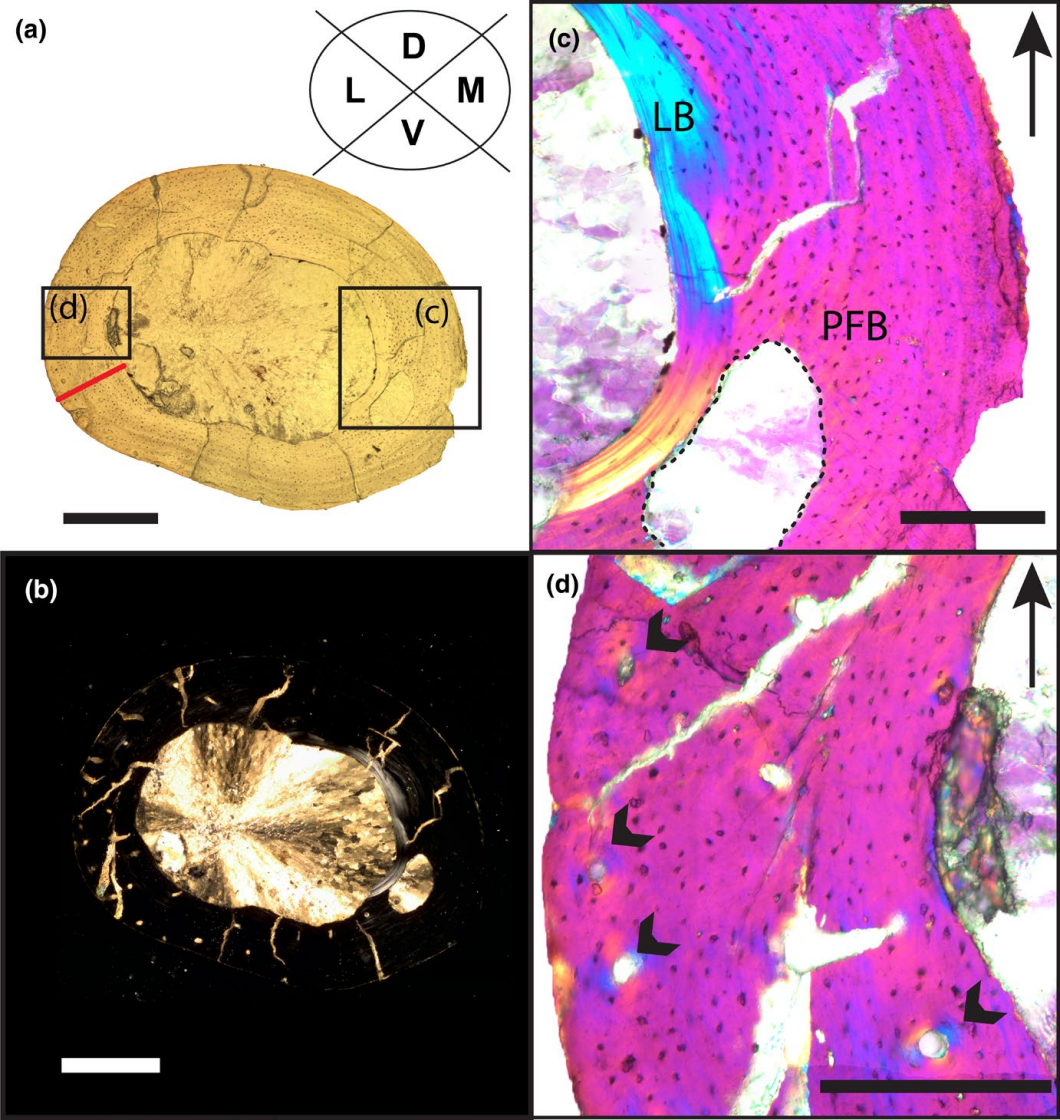
Osteohistology of PEFO 45274 in (a) plane polarized and (b) cross‐polarized light (scale bars = 500 μm). (c) Endosteally deposited lamellar bone (LB) shown in cross‐polarized light with a gypsum wave plate (scale bar = 200 μm). (d) Close‐up in cross‐polarized light with a gypsum wave plate showing primary osteons and simple vascular canals indicated by the black arrows (scale bar = 200 μm). Arrows with lines point dorsally. Red line represents the transect used in growth rate calculations. Dotted line outlines missing bone. D, dorsal quadrant; L, lateral quadrant; M, medial quadrant; PFB, parallel‐fibered bone; V, ventral quadrant.

The cross‐section of PEFO 45274 is sub‐circular to oval shaped with an open medullary cavity free of trabecular or coarse cancellous bone (Figure  4a,b). In the dorsal and medial quadrants, the endosteal margin preserves avascular lamellar bone (Figure 4c) as highly organized collagen fibrils visible in cross‐polarized light (Figure 4c,d). The endosteal margins of the ventral and lateral quadrants do not possess any lamellar bone, erosional cavities, or secondary osteons that would suggest resorption and/or expansion of the medullary cavity. Throughout the inner and outer cortex, PEFO 45274 preserves parallel‐fibered bone that is mostly avascular with a scattering of few primary osteons (~5 canals/mm^2^; Figure 4d) surrounding simple vascular canals and arranged longitudinally. Secondary osteons are not observed in any area of the cortex. Osteocyte lacunae are varied in shape with globular/round, flattened, and oval‐shaped morphologies present throughout the cortex in a random distribution except within the endosteally deposited lamellar bone, where osteocyte lacunae are flattened parallel to the periosteal and endosteal margins. In some regions of the cortex, osteocyte lacunae may be parallel, perpendicular, and oblique with respect to the periosteal surface with no clear preferred arrangement.

Throughout the cortex, there are no LAGs or annuli, nor is there evidence of a hatching line. In plane polarized light, there appear to be dark linear features that run parallel to the periosteal surface in the dorsomedial and ventral regions of the cross‐section but do not trace the entire cortex. However, these features do not appear in cross‐polarized light (Figure S1) nor are birefringent in cross‐polarized light (i.e., remain dark) with a gypsum wave plate (Figure S2). We argue that the linear features observed in plane polarized light may be diagenetic staining as opposed to a true histological feature and that this individual did not deposit LAGs or annuli at any point in life.

### 3.4 Growth rate calculation

The measured transect (Figure 4a) of 378.5 μm gives a range of appositional growth rates of approximately 1.0 μm/day (no growth hiatus), 1.3 μm/day (90‐day hiatus), and 2.0 μm/day (half‐year hiatus). Our values are within the lower end of the 95% confidence intervals of the femoral growth rates obtained by Cubo et al. (2012) using a phylogenetic estimation method for the aetosaur *Typothorax*, a phytosaur (“*Rutiodon*”), and those measured experimentally by the same authors for some extant squamates (*Lacerta vivipara*, *Podarcis muralis*) and a turtle (*Pelodiscus sinensis*). The growth rates for PEFO 45274 are lower than those of all other archosaur, varanid, and mammal femora studied by Cubo et al. (2012). The rates for PEFO 45274 are also on the low end of the range of measured growth rates for parallel‐fibered bone with longitudinal vascular canals obtained by de Margerie et al. (2002) for mallard duck (*Anas platyrhynchos*) long bones, which may be expected due to the much more rapid growth and maturation of extant Aves compared to that of extinct pseudosuchians and non‐archosaurian archosauriforms (Cubo et al., 2012). Even if we assume this rate is an underestimate due to the medullary cavity erosion of embryonic bone and bone deposited during some portion of its first year, the estimated daily growth rate of PEFO 45274 is comparable to or lower than that of related diapsids. Therefore, we conclude it is plausible, but not certain, that this individual could have deposited the preserved thickness of cortical bone within a single year, congruent with our hypothesis of its sub‐yearling age derived from its small size and external features suggestive of an early ontogenetic stage.

### 3.5 Body size estimation

The total body length estimates of PEFO 45274 and PEFO 45199 are 457.1 mm and 509.5 mm, respectively, approximately twice as long as hatchling individuals of extant *A. mississippiensis* (Erickson, 2005; Ikejiri, 2012). Additionally, the SVLA estimates of PEFO 45274 and PEFO 45199 are 178.08–232.60 mm and 250.5 mm, respectively, which also are approximately 60–200% longer than those of hatchling *A. mississippiensis* (i.e., ~110 mm; Congdon et al., 1995). The BM estimates of PEFO 45274 and PEFO 45199 are 101.6–248.7 g and 317 g, respectively, which is significantly larger than what is reported for hatchling alligators (BM ≈ 45–50 g; e.g., Millnes et al., 2001; MOR‐OST‐1647 [Table S2]). Under the growth model proposed by Jacobsen and Kushlan (1989) for *A. mississippiensis* in the Florida Everglades, individuals with an SVL greater than 180 mm are older than 1 year, which is inconsistent with the size‐age relationship observed for the phytosaur femora in this study that suggests a less than one‐year‐old phytosaur has an SVLA that ranges between ~180–235 mm. Despite being approximately the same size as a 1–2‐year‐old alligator, osteohistological evidence from PEFO 45274 does not support that age determination because there is no evidence of any LAGs, annuli, or other indicators of cyclical growth (i.e., alternating patterns of tissue deposition [e.g., alternation of high‐ versus low‐organized PFB; Teschner, Konietzko‐Meier, & Klein, 2022]) in the thin section. Although it is possible that an annulus or LAG may have been destroyed by medullary expansion, the histology as preserved may suggest this individual, despite its size, might not have reached one full year of age. The discrepancies revealed here may suggest that growth strategies between *Alligator* and phytosaurs are not as comparable as one may suspect given their superficial morphological resemblance, and those distinctions may be driven by phylogenetic or ecological/environmental differences.

## 4 DISCUSSION

### 4.1 Phytosaur growth dynamics

Phytosaurs have long been considered “crocodylian‐like” (Buffetaut et al., 1988; de Ricqlès et al., 2003; Camp, 1930; Gregory, 1962; Hoffman et al., 2021; Hunt et al., 2006; Renesto & Lombardo, 1999) and assumed to grow slowly, despite differences in skeletal anatomy and phylogenetic relationships. Even though previous researchers attempted to provide additional histological insight into the growth dynamics at the base of the archosaur tree and its stem (e.g., Cubo et al., 2012; de Ricqlès et al., 2003, 2008; Legendre et al., 2016; Werning, 2013; Werning & Nesbitt, 2016), those studies were not able to include phytosaurs within this ontogenetic and systematic context because of the lack of small, taxonomically identifiable phytosaur postcranial material. As a result, some studies (Cubo et al., 2012; de Ricqlès et al., 2003; Legendre et al., 2016) only included data from a single specimen (UCMP 25921, assigned to *Rutiodon*, and later, determined to be an indeterminate phytosaur [Werning, 2013]) and do not account for any potential extrinsic influences on growth, species‐specific growth rates, or developmental plasticity that may be occurring at the base of the archosaur tree, thus confounding our understanding of the evolution of growth across Archosauria.

**FIGURE 5:**
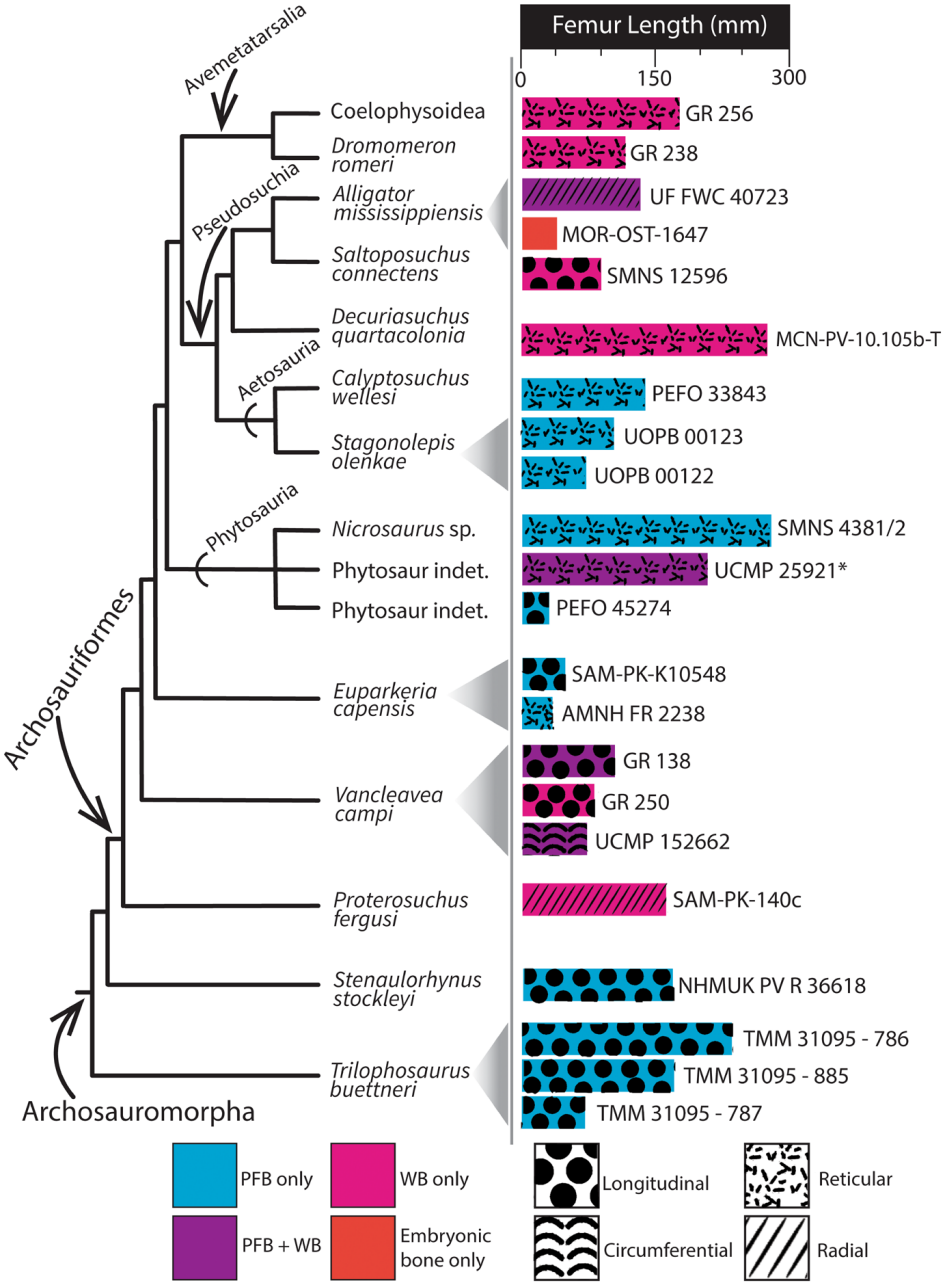
Cladogram of select taxa used for comparative histology among archosauromorphs showing histological variation in bone tissue organization and vascularity with respect to size. The data underlying this figure are available in S1. Cladogram modified from Nesbitt (2011) and Ezcurra (2016). *femur length calculated from estimated femoral diameter. PFB, parallel‐fibered bone; WB, woven bone.

The preservation of nearly avascular parallel‐fibered bone in a small, ontogenetically immature phytosaur is unexpected. However, in a similarly tiny aetosaur humerus (SMNS 5570–21, *Aetosaurus ferratus* [Teschner, Konietzko‐Meier, Desojo, et al., 2022]), parallel‐fibered bone was the dominant tissue and may suggest that slow growth during the earliest ontogenetic stages of large‐bodied Late Triassic taxa is more common than assumed. Classically, skeletal age models are based on the concept that an organism demonstrates linear growth rates that decrease with age and plateau once maximum body size is attained (e.g., Woodward et al., 2013). Therefore, we would assume that smaller, skeletally immature individuals will possess faster growth rates than their larger‐bodied skeletally mature counterparts. Because a North American large‐bodied phytosaur (i.e., FL > 200 mm) from the Upper Blue Mesa Member *Placerias* Quarry (UCMP 25291, Phytosauria indet., Werning, 2013) preserves woven bone with anastomosing vascular canals (Figure 5; de Ricqlès et al., 2008; Werning, 2013), we would expect that a skeletally immature phytosaur individual from the same stratigraphic interval would preserve bone tissues and vascular characteristics supporting faster growth based on the growth curves and models that have been produced for archosaur taxa (de Buffrénil et al., 2021; Erickson, 2005; Erickson & Druckenmiller, 2011; Lee et al., 2013; Taborda et al., 2013; Tumarkin‐Deratzian, 2007; Woodward et al., 2013). However, based on the overall small femoral size, lack of a hatching line or any LAGs in PEFO 45274, the flatter proximal margin, and lack of an anteromedial tuber on the proximal head in PEFO 45274 and PEFO 45199 in comparison to other larger phytosaur femora, we interpret this specimen as a slow‐growing small phytosaur that may have died within the first year of life.

Even though the lack of a hatching line might suggest that PEFO 45274 is an embryonic individual, the lack of any woven, disorganized, or well‐vascularized embryonic bone tissue and the presence of endosteally deposited lamellar bone suggest that the femur of this individual had already destroyed any embryonic bone via cortical drift and prior expansion of the medullary cavity. It may be possible that the lack of a hatching or neonatal line could be due to similar growth rates between *in ovo* (or in utero in viviparous taxa) and post‐hatching stages; however, we use the presence of endosteal lamellar bone to refute claims that this individual may be embryonic because these bone tissues (=endosteal lamellar bone) have yet to be reported from either known embryos (Horner et al., 2001; Hugi & Sanchez‐Villagra, 2012; Nacarino‐Meneses & Köhler, 2018) or individuals hypothesized to preserve embryonic bone (Barta & Norell, 2021; Horner et al., 2001). Additionally, endosteally deposited lamellar bone signifies that the medullary cavity has ceased expansion (Chinsamy‐Turan, 2005) and instead, is depositing lamellar bone to account for cortical drift (Enlow, 1962), which is surprising in an inferred skeletally immature individual because medullary expansion typically occurs during early ontogeny (Chinsamy‐Turan, 2005). Therefore, it is possible that medullary expansion stopped earlier than is typically observed across archosaur histology and phytosaurs underwent remodeling at earlier ontogenetic stages than expected.

At less than 1 year of age, we estimate PEFO 45274 to be about the size of a 1.5 to 2‐year‐old individual of *A. mississippiensis* (Collett, 2023; Jacobsen & Kushlan, 1989), which may suggest that phytosaurs hatched at larger body sizes than hatchlings of *A. mississippiensis*. If PEFO 45274 hatched at the same size as a hatchling *A. mississippiensis* (SVL ≈ 110–150 mm based on Jacobsen & Kushlan, 1989), then this individual would have needed to have doubled in total length in less than 1 year and experienced rapid growth in the first year of life, in contrast to what is observed in the osteohistology. In fact, the low vascularity preserved in PEFO 45274 likely is attributable to its relatively small size, similarly to what has been observed in small‐bodied sauropsids (Cubo et al., 2005) and *Archaeopteryx* (Erickson et al., 2009). In these smaller‐bodied organisms, bone tissues were mostly avascular and were able to attain necessary nutrients via the periosteum or endosteum (Montes et al., 2010).

Phytosaur femoral osteohistology suggests faster growth rates at larger body sizes and hypothesized later ontogenetic stages in comparison to PEFO 45274 due to the preservation of woven bone (specifically in North American taxa [de Ricqlès et al., 2003; Werning, 2013]) and greater vascular disorganization (i.e., radial, reticular, or plexiform vascular patterns), and osteon density across all other phytosaur individuals (de Ricqlès et al., 2003; Teschner, Konietzko‐Meier, & Klein, 2022; Werning, 2013). Some previous studies of phytosaur femoral histology (de Ricqlès et al., 2003; Werning, 2013) revealed that, in circumstances where secondary remodeling does not obscure the primary growth signal, fast‐growing vascular patterns (i.e., “sub‐plexiform” sensu de Ricqlès et al., 2003) within a fibrolamellar bone complex may be preserved in the inner cortex, and periosteally, the bone tissue structure will transition to slow‐growing vascular patterns within parallel‐fibered or avascular lamellar bone (de Ricqlès et al., 2003). It is important to note that in both of those studies, UCMP 25291 was analyzed to describe phytosaur growth despite its elusive classification as either *Rutiodon* sp. (de Ricqlès et al., 2003) or an indeterminate phytosaur (Werning, 2013). Therefore, whether the histological properties reflect phylogenetic differences or extrinsic growth signal remains unknown.

However, not all phytosaur femoral histology preserves this expected fast‐to‐slow growth rate pattern. Osteohistology conducted on a femur of a mystriosuchin phytosaur from the Totes Gebirge in Austria, *Mystriosuchus steinbergi*, revealed that the entire cortex was composed of parallel‐fibered bone with radially anastomosing longitudinal vascular canals (Butler et al., 2019), suggesting overall slow growth rates that were maintained throughout growth. Again, it is important to note that *Mystriosuchus*, including *M. steinbergi*, is considered to be marine (Butler et al., 2019), and that the preserved osteohistological differences between *M. steinbergi* and other phytosaurs may be attributable to its divergent paleoecology. Additionally, a population of *Parasuchus* cf. *P. arenaceus* (=*Paleorhinus* cf. *P. arenaceus* sensu Dzik & Sulej, 2007) from the Late Triassic Krasiejów locality in Poland and an individual of *Nicrosaurus* from Germany were found to possess only parallel‐fibered bone that alternated between high and low degrees of organization with randomly spaced annuli (and a LAG in the *Nicrosaurus* femur) and deviated overall from the standard pattern that reflects fast growth during early ontogeny that slows during later ontogenetic stages due to extrinsic effects (e.g., seasonality) on growth (Montes et al., 2010; Teschner, Konietzko‐Meier, & Klein, 2022). Additionally, the vascularity of bone is highly dependent on bone size (i.e., volume) because larger bones require a larger blood supply and therefore, increased degrees of vascularization (Cubo et al., 2005). As a result of the evolution of larger body sizes, increased vascularity may be produced as a result of correlative selection (Cubo et al., 2005). Our histological findings suggest that phytosaurs may exhibit size‐dependent growth through ontogeny (Figure 5) that is most evident in North American phytosaur taxa, and that increased vascularity may be attributable to positive selection for faster growth rate and larger size (Cubo et al., 2005; Montes et al., 2010). Despite the overall small set of osteohistological properties that can be compared across known samples, the comparable properties are quite varied, and the studies conducted thus far have likely yet to broach the true extent of histological variation preserved across taxa. Therefore, phytosaurs likely possessed growth patterns more complex than previously considered, with differences that may be attributable to developmental plasticity, ecology, environment, or functional constraints based on bone size.

### 4.2 Phytosaur growth dynamics within a phylogenetic context

In comparison to other archosauromorphs, PEFO 45274 preserves histological patterns somewhat similar to those of non‐archosaurian archosauriforms (Figure 5) such as *Euparkeria capensis* (Botha‐Brink & Smith, 2011; Werning, 2013) and non‐archosauriform archosauromorphs such as *Trilophosaurus buettneri* (Werning, 2013) and rhynchosaurs (i.e., *Stenaulorhychus stockleyi*; Werning & Nesbitt, 2016). Similar to the diminutive PEFO 45274 (est. FL = 30 mm), the femora of *E. capensis* (est. FL = 45–70 mm), *T. buettneri* (est. FL = 75–220 mm), and *S. stockleyi* (FL = 180 mm) were reported to preserve low to moderate vascular density, with simple longitudinal vascular canals in parallel‐fibered bone (Botha‐Brink & Smith, 2011; Werning, 2013; Werning & Nesbitt, 2016). The osteohistological data from those taxa represent relatively slower growth patterns compared to those of archosauriforms (Figure 5) such as *Vancleavea campi* (e.g., GR 250 and UCMP 152662; Werning, 2013) and *Proterosuchus fergusi* (e.g., SAM‐PK‐K140c; Botha‐Brink & Smith, 2011), which preserve woven, fibrolamellar bone with anastomosing and longitudinally oriented vascular canals. The shared, parallel‐fibered, low vascularity bone tissue among the most skeletally immature (i.e., smallest) leptosuchomorph phytosaurs (i.e., FL < 50 mm) and non‐archosaur archosauromorphs like *E. capensis, T. buettneri*, and *S. stockleyi* may be plesiomorphic for Archosauromorpha, and subsequent evolution of fast‐growing bone in some archosauriforms may have developed as a result of the evolution of larger body sizes.

Non‐archosaurian archosauriforms have been inferred to possess more rapid ancestral growth and metabolic rates than living pseudosuchians, which are hypothesized to have secondarily slowed growth and metabolism to accommodate a semi‐aquatic ecology (Botha et al., 2023; Cubo et al., 2012; Legendre et al., 2016). Phylogenetic comparative methods using osteocyte density as a proxy for metabolic rates were instrumental in determining the ancestral growth rate for archosauriforms and indicate that metabolic rates in *E. capensis* far exceed those of *P. fergusi* (Legendre et al., 2016) despite histological findings of parallel‐fibered and fibrolamellar bone tissue, respectively (Botha‐Brink & Smith, 2011). That discrepancy suggests that there may be a large amount of histological variation that is not being considered in investigations of the evolution of growth in archosauromorphs. For example, Botha‐Brink and Smith (2011) were able to conduct osteohistological analyses of only three individuals of *E. capensis* with skull lengths between 88 and 96% of the largest known specimens. Therefore, the earliest ontogenetic stages of *E. capensis* have yet to be histologically sampled and may preserve evidence of the high ancestral growth rate hypothesized for archosauriforms. Because of the influence of size and ontogenetic stage on histological features preserved in bone, we recommend, when possible, to compare the growth dynamics across similarly sized individuals or similar ontogenetic stages.

A neonate *A. mississippiensis* of unknown wild or domestic origin (MOR‐OST‐1647; estimated TL ≈ 390 mm; Bailleul et al., 2016; Woodward et al., 2014) similar in body size to PEFO 45274 preserves radically different cross‐sectional histology (MorphoBank Project 731; Woodward et al., 2014) in comparison to that of PEFO 45274. Whereas PEFO 45274 preserves relatively thick cortical bone and few vascular canals, MOR‐OST‐1647 has thin, embryonic bone with a higher degree of vascularity (Woodward et al., 2014), which may have been influenced by extrinsic influences given that we are unsure whether this individual is captive‐bred or wild caught. Another slightly larger specimen of *A. mississippiensis* (MOR‐OST‐1648; estimated FL ≈ 63 mm) was reported to have both parallel‐fibered and woven bone tissue types with higher degrees of vascularity that are typically associated with crocodylian growth indicated by radially anastomosing longitudinal canals (Woodward et al., 2014). A different *A. mississippiensis* specimen, MOR‐OST‐1648, (TL ≈ 950 mm; Woodward et al., 2014) preserves at least two LAGs, a partial annulus, and lamellar bone that surrounds the entire endosteal margin (Woodward et al., 2014), features that are not present in PEFO 45274 with the exclusion of the presence of endosteal lamellar bone. However, in PEFO 45274 the endosteal lamellar bone does not surround the entire medullary cavity in contrast to what is observed in MOR‐OST‐1648. Interestingly, a portion of the inner cortex (between the endosteal lamellar bone and first partial annulus) of MOR‐OST‐1648 was reported to possess parallel‐fibered bone and lower vascular density in comparison to the remainder of the cortex (Woodward et al., 2014), which may suggest that the histological patterns in extant hatchling crocodylians are also impacted by size, similar to what is seen in PEFO 45274. This finding was only reported in one of the *A. mississippiensis* femora of this growth series suggesting that the transition from low‐to‐high vascular density observed in MOR‐OST‐1648 is not common amongst skeletally immature crocodylians. The differences in vascular patterns and density between PEFO 45274, MOR‐OST‐1647, and MOR‐OST‐1648 suggest that in the earliest post‐hatching growth stages, phytosaurs were likely growing slower than what is observed in living crocodylians such as *A. mississippiensis* (Jacobsen & Kushlan, 1989; Tumarkin‐Deratzian, 2007; Woodward et al., 2011, 2014), *Crocodylus niloticus* (Audije‐Gil et al., 2023), *Caiman latirostris* (Mascarenhas‐Junior et al., 2021; Pereyra et al., 2024), and *Caiman yacare* (de Andrade et al., 2020), or extinct taxa such as *Neuquensuchus universitas* (non‐neosuchian Crocodyliformes; Garcia Marsà et al., 2022) that all have evidence of fast‐growing woven bone tissues.

It is important to note that even though all living crocodylians are regarded as typical exemplars of taxa with ectothermic growth (de Ricqlès et al., 2003), growth dynamics across extinct and extant pseudosuchians are likely diverse. As noted above, extant and extinct crocodylians have the ability to grow fast and lay down the associated woven bone tissues with anastomosing vascular canals. However, exclusively slow‐growing bone tissues such as low‐vascularity parallel‐fibered bone have also been reported in some extant (e.g., *Crocodylus niloticus* [Audije‐Gil et al., 2023]) and extinct (e.g., *Ibersosuchus macrodon* [Cubo et al., 2017], *Susisuchus anatoceps* [ulna and rib; Sayão et al., 2016]) taxa. Diverse growth strategies additionally exist among non‐crocodylian pseudosuchians. In the 'rauisuchians' (a grade of early‐branching Loricata sensu Nesbitt & Desojo, 2017), growth was classified as fast, evident from the appearance of woven bone arranged in a fibrolamellar complex during the first years of growth that shifted to slow‐growing parallel‐fibered bone later in ontogeny (de Farias et al., 2023; de Ricqlès et al., 2003; Klein et al., 2017; Ponce et al., 2017), consistent with what is classically reported for most vertebrates (de Buffrénil et al., 2021; Huttenlocker et al., 2013; Lee et al., 2013; Woodward et al., 2013). This pattern is shared among some aetosaurs (e.g., *Calyptosuchus* sp. [de Ricqlès et al., 2003] and *Aetosauroides scagliai* [Ponce et al., 2023]). However, femoral histology of *Stagonolepis olenkae* from the Krasiejów locality in Poland revealed only parallel‐fibered bone with simple vascular canals arranged within a longitudinal or reticular network with highly irregularly spaced growth zones that do not conform to the expected fast‐to‐slow growth pattern potentially due to extrinsic influences on growth (Teschner, Konietzko‐Meier, & Klein, 2022). Additionally, histology of the inferred hatchling *A. ferratus* suggests that aetosaurs may similarly preserve slow growth rates during the earliest stages of ontogeny (Teschner, Konietzko‐Meier, Desojo, et al., 2022) and highlights the variable growth across archosauriforms through ontogeny that may be resultant from plasticity or ecological/environmental influence on growth. Despite this diversity of growth patterns observed across pseudosuchians, only a single species (*A. ferratus*) has mirrored the phytosaur growth pattern observed in this study that shows that the smallest, ontogenetically immature specimens preserve slower growth rates than larger, skeletally mature individuals.

To account for the range and variation of histological features concerning size and ontogeny, we recommend more extensive osteohistological investigations across various size classes (and inferred ontogenetic stages) of non‐archosaurian archosauriforms (e.g., phytosaurs, *E. capensis, P. fergusi*) and non‐archosauriform archosauromorphs (e.g., rhynchosaurs), which may reveal whether their osteohistological properties are evidence of shared phylogenetic growth regimes, developmental plasticity, or size‐dependent growth. Additionally, we suggest that studies reporting femoral histological data share size data using measurements from Farlow et al. (2005) and Campione & Evans (2012)  (see Table S1) and report the maximum diaphyseal circumferences so that size‐dependent growth may also be accounted for in comparisons across taxa. Considering femoral lengths in evolutionary studies of growth has proven useful in determining ancestral growth conditions in avialans (Erickson et al., 2009), but that type of osteohistological study has yet to be conducted for early archosauriforms and archosauromorphs due to the lack of growth series. By doing so here, we reveal a previously unknown ontogenetic stage of a phytosaur growth trajectory and provide a basis to compare growth trajectories across archosauriforms to determine the ancestral growth condition in Archosauria.

### 4.3 Presence of hatchling phytosaurs in North America

The presence of PEFO 45274 and PEFO 45199 confirms the preservation of possible hatchling phytosaurs in North America. If these individuals represent hatchling‐age phytosaurs, then it is possible that the PFV 456 bonebed may have formed near the location where phytosaurs were nesting and/or where hatchling individuals were living. It is unlikely that the delicate bones of PEFO 45199 and PEFO 45274 were transported very far due to the lack of abrasion (Rogers et al., 2007; Kligman, 2023). The absence of similarly tiny‐sized phytosaur elements from the many phytosaur‐bearing bonebeds across Late Triassic Pangaea suggests that the PFV 456 bonebed may capture a unique depositional setting that accumulated and preserved these bones, or more probably, that tiny phytosaur elements like these have been overlooked, misidentified, or destroyed by collecting procedures in other phytosaur‐bearing bonebeds. Ultimately, this study highlights the biological significance of implementing microvertebrate fossil collection procedures (e.g., Kligman, 2023; Kowalski et al., 2019) to enrich the fossil record with the smallest members of paleoecosystems, allowing for novel investigations of ontogeny, body size, and paleodiversity.

## 5 CONCLUSIONS

PEFO 45274 represents the smallest and ontogenetically youngest phytosaur (potentially less than 1 year old) specimen known. This specimen does not display histological markers suggestive of faster growth in the earliest stages of ontogeny similar to what is observed in the (small‐sized and skeletally immature) humeri of the aetosaur *Aetosaurus ferratus* from the German Kaltental locality and in contrast to what is observed in most extinct and extant crocodylians, as well as throughout all growth stages of dinosaurs and other avian‐line archosaurs. However, faster growth rates may occur in phytosaurs during slightly later ontogenetic stages (e.g., after two or more years of life), suggesting that phytosaurs may exhibit size‐dependent growth through ontogeny and that histological features associated with fast growth may result from correlative selection at larger sizes. The osteohistology of this earliest known ontogenetic stage of a phytosaur does not mirror what is observed in most neonate or hatchling extant crocodylian bones, suggesting that crocodylians and phytosaurs experience different growth patterns throughout ontogeny. This sampled phytosaur specimen grew most similarly to non‐archosaurian archosauromorphs (e.g., *T. buettneri* and *S. stockleyi*) and smaller archosauriforms such as *E. capensis* and *V. campi* based on the presence of parallel‐fibered bone with low vascular density that is arranged longitudinally. However, it is unknown whether all of the smallest individuals of these other taxa would exhibit a similar osteohistological pattern or if this is unique to phytosaurs. When factoring in size, new growth patterns are revealed in Phytosauria that are unexpected and potentially unique, suggesting that accounting for size in understanding the variation of histological patterns in archosauriforms may highlight new trends in the evolution of growth across Archosauria.

## AUTHOR CONTRIBUTIONS

ERG conceived the project, performed all analyses and body size calculations, wrote the manuscript, prepared the figures, and reviewed drafts of the paper. DEB conducted growth rate calculations, helped analyze and interpret the data, write and edit the manuscript, and review drafts of the paper. BTK collected the material, facilitated specimen acquisition, helped write and edited the manuscript, and reviewed drafts of the paper. SJN helped analyze and interpret the data, edit the manuscript, and review drafts of the paper. ADM collected the material, facilitated specimen acquisition and permissions for destructive analysis, reviewed drafts of the paper, and edited the manuscript. WGP facilitated specimen acquisition, permissions for destructive analyses, edited the manuscript, and reviewed drafts of the paper. MRS supervised the study, provided access to the loaned specimen, edited the manuscript, and reviewed drafts of the paper.

## Supporting information


Data S1.

Appendix S1.

Data S2.

Data S3.

## Data Availability

The data supporting the findings of this study are available within the article and its supplementary materials. High resolution images and 3D models are available for download upon request in Morphosource.com (Project ID #000628894).
