# Supplementary material for: Osteohistological signal from the smallest known phytosaur femur reveals slow growth and new insights into the evolution of growth in Archosauria
Source: J Anat. 2024 Dec 3;247(3-4):556–75. doi: 10.1111/joa.14185 (PMC12397225; doi:10.1111/joa.14185)
Supplement: Supplementary file 2 — Appendix S1. [file JOA-247-556-s001.pdf]

Supplementary Figures and Tables:

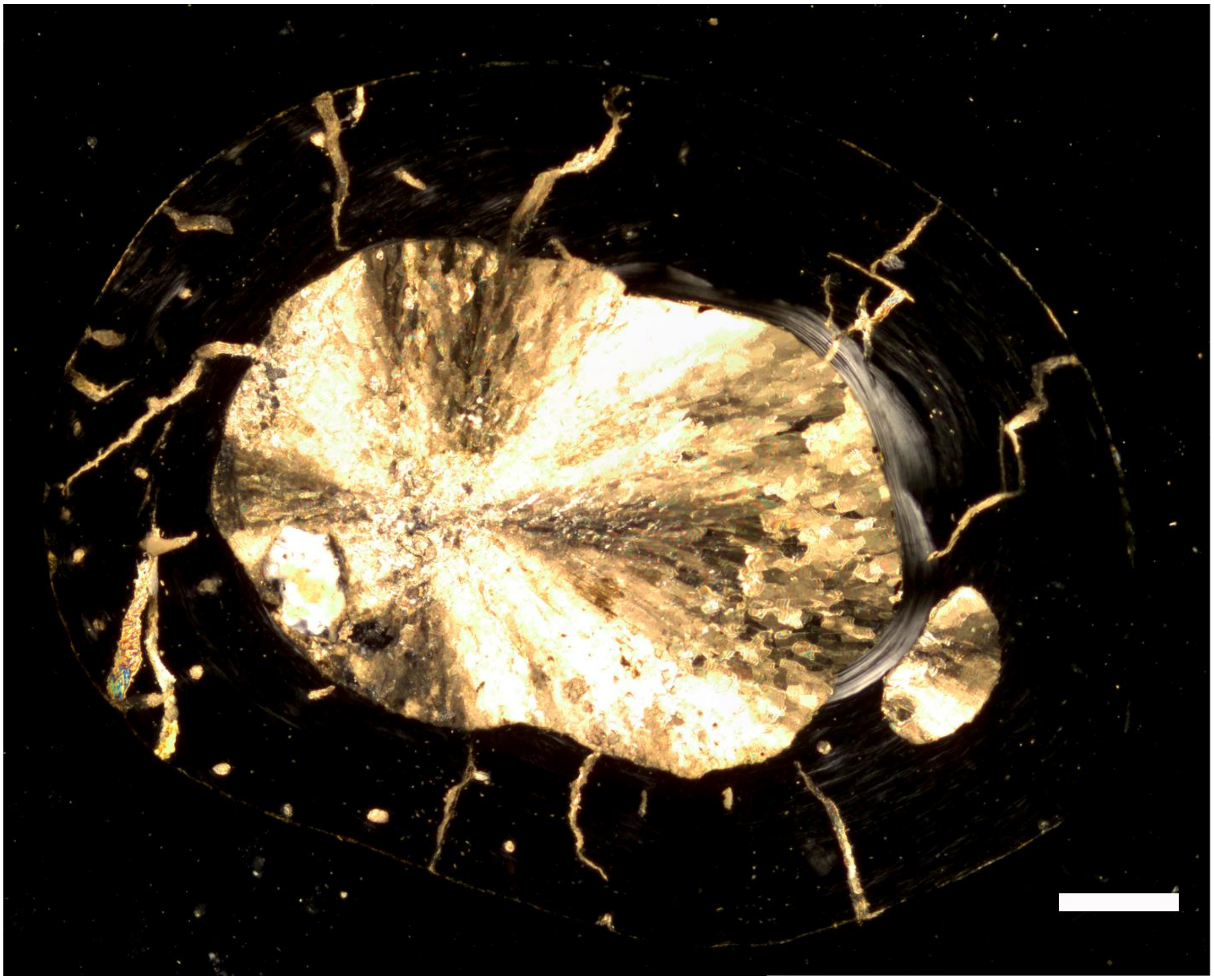

S1. PEFO 45274 femoral osteohistology in cross-polarized light. Scale bar = 250 μm.

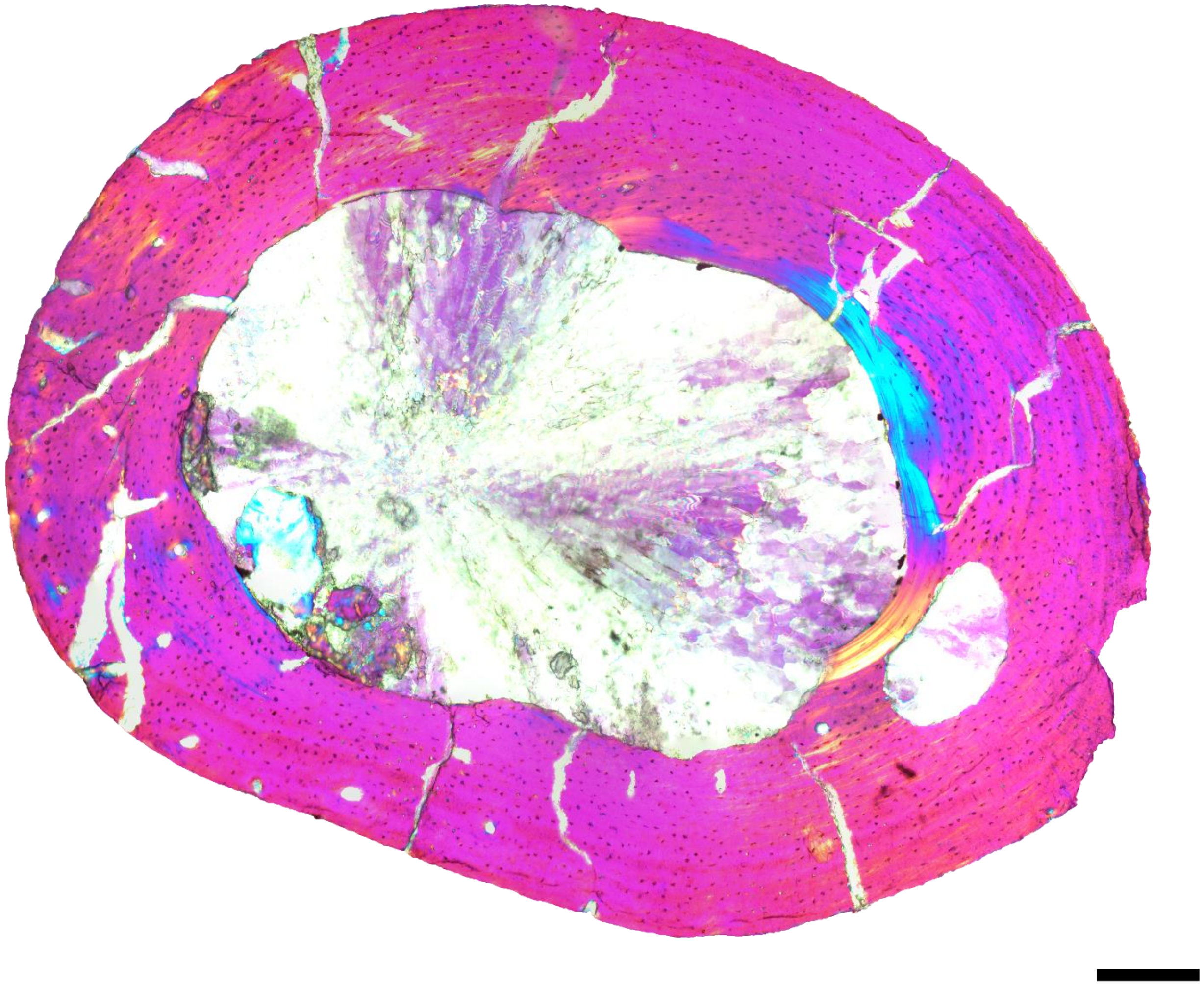

S2. PEFO 45274 femoral osteohistology in cross-polarized light with a lambda wave plate. Scale bar = 250 μm.

Supplementary Table 1. Femoral lengths and cross-sectional dimensions of taxa compared in this study. MSC = Mid-shaft circumference; MMSW = Maximum mid-shaft width. ? = not measured; NA = not applicable/not able to be measured.

Clade	Taxon	Specimen Number	FL (mm)	est. FL° (mm)	est. FL **	MSC (mm)	MMSW (mm)
Phytosauria	Phytosauria indet.	**PEFO 45199**	34.12	36.76	–	11.00	NA
		**PEFO 45247**	?	31.64	24.02	*6.47*	2.14
		UCMP 25921	245.47	NA	NA	NA	30.00
	Mystriosuchus steinbergi	NHMW 1986/0024/0013	250.00	NA	NA	?	31.10
	Parasuchus cf. *arenaceus*	UOBS 03370	390.00	NA	NA	?	40.00
	Parasuchus cf. *arenaceus*	UOBS 01026	370.00	NA	NA	?	35.00
	Parasuchus cf. *arenaceus*	UOBS 00143	250.00	NA	NA	?	33.00
	Nicrosaurus sp.	SMNS 4381/2	295.00	NA	NA	?	42.00
Archosauriformes	*Euparkeria capensis*	AMNH FARB 2238	?	NA	46.05	13.90	5.30
		SAM-PK-K10010a	?	NA	?	?	6.10
		SAM-PK-K10548	68.00	NA	NA	?	7.20
	Proterosuchus fergusi	SAM-PK-K140c	167.00	NA	NA	?	19.70
		SAM-PK-11208a	?	NA	?	?	?
	Vancleavea campi	GR 138	*73.00*	NA	NA	?	?
		GR 250	?	NA	82.71	27.66	8.49
		UCMP 152662	?	NA	81.95	27.36	8.66
Archosauromorpha	*Trilophosaurus buettneri*	TMM 31025 - 787	?	NA	69.15	22.41	7.13
		TMM 31025 - 1064	?	NA	84.04	28.18	8.80
		TMM 31025 - 1063	?	NA	108.63	38.10	12.50
		TMM 31025 - 885	?	NA	143.62	52.90	17.93
		TMM 31025 - 786	?	NA	207.47	81.50	22.96
	Stenaulorhynus stockleyi	NHMUK PV R 36618	182.00	NA	NA	102.00	33.65
Pseudosuchia	*Alligator mississippiensis*	MOR-OST-1647	?	NA	25.69	*4.70*	?
		MOR-OST 1648	?	NA	58.66	18.47	?
		MOR-OST 1649	?	NA	79.04	26.22	?
		MOR-OST 1650	?	NA	84.85	28.50	?
		UF FWC 40723	134.77	NA	134.33	48.90	16.20
	Revueltosaurus callenderi	PEFO 33843	?	NA	116.17	41.23	15.07
	Aetosauria indet.	UCMP 25914	?	NA	NA	?	?
	Stagonolepis olenkae	UOPB 00123	100.00	NA	NA	?	66.00
	Stagonolepis olenkae	UOPB00122	89.00	NA	NA	?	65.00
	Saltoposuchus	SMNS 12596	107.00	NA	NA	NA	NA
Avemetatarsalia	*Dromomeron romeri*	GR 221	?	NA	84.06	28.19	9.17
	Coelophysoidea	GR 256	?	NA	174.21	66.37	20.81

Supplementary Table 2. Estimated total lengths (TL), anterior snout-vent lengths (SVLA), body masses (BM), reported blood tissue types, and vascular arrangements of taxa compared in this study. PFB= Parallel-fibered bone; WB = Woven bone; EB = Embryonic bone; L= Longitudinal; C=Circumferential; Ra=Radial; Re=Reticular; P=Plexiform; NA = Not applicable.

Clade	Taxon	Specimen Number	est. TL* (mm)	est. TL° (mm)	est. TL (mm)	est BM (kg)	SVLA (mm)	Bone Tissue Type	Vascular arrangment
Phytosauria	Phytosauria indet.	**PEFO 45199**	494.48	NA	535.09	0.32	250.47	**?**	**NA**
		**PEFO 45247**	NA	458.61	348.60	0.10-0.25	178.08-224.44	PFB	L
		UCMP 25921	245.47	NA	NA	NA	NA	PFB+WB	P
	Mystriosuchus steinbergi	NHMW 1986/0024/0013	3536.45	NA	NA	205.14	1798.33	PFB	L,Re
	Parasuchus cf. *arenaceus*	UOBS 03370	5516.05	NA	NA	872.89	2802.13	PFB	L,Re,P
	Parasuchus cf. *arenaceus*	UOBS 01026	5233.25	NA	NA	735.62	2658.73	PFB	L,Re,P
	Parasuchus cf. *arenaceus*	UOBS 00143	3536.45	NA	NA	205.73	1798.33	PFB	L,Re,P
	Nicrosaurus sp.	SMNS 4381/2	4172.75	NA	NA	352.31	2120.98	PFB	L,Re
Archosauriformes	*Euparkeria capensis*	AMNH FARB 2238	NA	NA	653.13	0.84	336.04	PFB	Re
		SAM-PK-K10010a	NA	NA	NA	NA	NA	PFB	L
		SAM-PK-K10548	962.97	NA	962.97	2.99	493.39	PFB	L
	Proterosuchus fergusi	SAM-PK-K140c	2362.83	NA	2362.83	55.44	1203.22	WB	L, Ra
		SAM-PK-11208a	NA	NA	NA	NA	NA	PFB	L
	Vancleavea campi	GR 138	1033.67	NA	NA	3.76	529.24	PFB+WB	L
		GR 250	NA	NA	401.14	5.65	598.90	WB	L, Re
		UCMP 152662	NA	NA	396.80	5.48	593.42	WB+PFB	L,C
Archosauromorpha	*Trilophosaurus buettneri*	TMM 31025 - 787	NA	NA	325.27	3.15	501.64	LB	L
		TMM 31025 - 1064	NA	NA	408.65	5.95	608.37	LB	L
		TMM 31025 - 1063	NA	NA	552.00	5.48	784.68	LB	L
		TMM 31025 - 885	NA	NA	765.86	33.96	1035.61	LB	L
		TMM 31025 - 786	NA	NA	1179.13	112.23	1493.40	LB	L
	Stenaulorhynus stockleyi	NHMUK PV R 36618	2574.93	NA	2574.93	73.32	1310.77	LB	L
Pseudosuchia	*Alligator mississippiensis*	MOR-OST-1647	NA	NA	265.93	0.04	137.06	EB	NA
		MOR-OST 1648	NA	NA	849.07	1.84	426.41	PFB+WB	L
		MOR-OST 1649	NA	NA	1143.52	4.87	572.52	PFB	L
		MOR-OST 1650	NA	NA	1227.50	6.14	614.19	PFB+WB	L, Ra
		UF FWC 40723	2030.00	NA	1942.49	27.32	968.96	PFB+WB	Re, Ra
	Revueltosaurus callenderi	PEFO 33843	NA	NA	1680.18	17.05	838.80	PFB	Ra
	Aetosauria indet.	UCMP 25914	NA	NA	NA	NA	NA	PFB+WB	Re
	Stagonolepis olenkae	UOPB 00123	1415.45	NA	1415.45	10.47	722.83	PFB	L, Re
	Stagonolepis olenkae	UOPB00122	1259.91	NA	1259.91	7.17	643.02	PFB	L, Re
	Saltoposuchus	SMNS 12596	1514.43	NA	1514.43	13.05	723.02	WB	L
Avemetatarsalia	*Dromomeron romeri*	GR 221	NA	NA	1216.14	5.96	608.55	WB	Re
	Coelophysoidea	GR 256	NA	NA	2518.72	63.60	1254.88	WB	Re
